# Glutathione S-Transferase Regulates Mitochondrial Populations in Axons through Increased Glutathione Oxidation

**DOI:** 10.1016/j.neuron.2019.04.017

**Published:** 2019-07-03

**Authors:** Gaynor A. Smith, Tzu-Huai Lin, Amy E. Sheehan, Wynand Van der Goes van Naters, Lukas J. Neukomm, Hillary K. Graves, Dana M. Bis-Brewer, Stephan Züchner, Marc R. Freeman

**Affiliations:** 1 Department of Neurobiology, University of Massachusetts Medical School, Worcester, MA 01605, USA; 2 Vollum Institute, Oregon Health & Science University, Portland, OR 97239, USA; 3 UK Dementia Research Institute, School of Medicine, Cardiff University, Cardiff CF24 4HQ, UK; 4 Molecular Biosciences, School of Biosciences, Cardiff University, Cardiff CF10 3AX, UK; 5 Department of Fundamental Neurosciences, University of Lausanne, 1005 Lausanne VD, Switzerland; 6 Department of Molecular and Human Genetics, Baylor College of Medicine, Houston, TX 77030, USA; 7 John P. Hussman Institute for Human Genomics, University of Miami, Miami, FL, USA; 8 Dr. John T. Macdonald Foundation Department of Human Genetics, University of Miami, Miami, FL, USA

**Keywords:** mitochondria, axons, neurons, glutathione, glutathione S-transferases, mitofusin, marf, redox, Gfzf, *Drosophila*

## Abstract

Mitochondria are essential in long axons to provide metabolic support and sustain neuron integrity. A healthy mitochondrial pool is maintained by biogenesis, transport, mitophagy, fission, and fusion, but how these events are regulated in axons is not well defined. Here, we show that the *Drosophila* glutathione S-transferase (GST) Gfzf prevents mitochondrial hyperfusion in axons. Gfzf loss altered redox balance between glutathione (GSH) and oxidized glutathione (GSSG) and initiated mitochondrial fusion through the coordinated action of Mfn and Opa1. Gfzf functioned epistatically with the thioredoxin peroxidase Jafrac1 and the thioredoxin reductase 1 TrxR-1 to regulate mitochondrial dynamics. Altering GSH:GSSG ratios in mouse primary neurons *in vitro* also induced hyperfusion. Mitochondrial changes caused deficits in trafficking, the metabolome, and neuronal physiology. Changes in GSH and oxidative state are associated with neurodegenerative diseases like Alzheimer’s. Our demonstration that GSTs are key *in vivo* regulators of axonal mitochondrial length and number provides a potential mechanistic link.

## Highlights

• A glutathione S-transferase (GST), Gfzf, decreases mitochondrial fusion in axons
• Gfzf loss causes hyperfusion through enhanced oxidation of glutathione
• GST loss or enhanced oxidation of glutathione increases the function of mitofusin
• Hyperfusion was associated with functional changes but not neurodegeneration

## Teaser

Smith and Lin et al. identify a glutathione S-transferase that decreases mitochondrial fusion in axons of neurons through unbiased genetic screening using *Drosophila*. *gfzf* mutants induce sustained hyperfusion through an enhanced oxidation of glutathione that increases the function of mitofusin.

## Introduction

Most neurons are generated during embryogenesis and are subsequently maintained throughout the entire life of an organism. Mitochondria are integral to sustaining neuronal health, and the long polarized processes of neurons pose a unique challenge for adequate mitochondrial positioning and maintenance. Cellular homeostasis and adequate ATP production is thought to be achieved in axons through regulation of several essential mitochondrial processes, including mitochondrial fusion, fission, biogenesis, degradation, and transport (Harbauer, 2017). The major pathways responsible for regulating these have been increasingly well described over the past decade and are highly conserved from humans to *Drosophila* (Hewitt and Whitworth, 2017, Vanhauwaert and Verstreken, 2015), but whether they uniformly regulate mitochondrial biology similarly in all neuronal compartments remains an open question.

Rapid and dynamic changes in mitochondrial length occur in response to the ever-changing environment of the cell (van der Bliek et al., 2013). Conditions that increase mitochondrial ATP consumption lead to enhanced fusion, allowing for the mixing of mtDNA and proteins, whereas metabolic signals that grossly uncouple the mitochondria may result in fusion inhibition and occur as a prerequisite to mitophagy and/or neurodegeneration (van der Bliek et al., 2013). Increased mitochondrial length can be classically achieved through increased function of the mitochondrial fusion proteins mitofusin (MFN) and optic atrophy 1 (OPA1) or decreased activity of the mitochondrial fission factors dynamin-related protein 1 (DRP1) and fission mitochondrial 1 (FIS1) (van der Bliek et al., 2013). The precise cellular processes that control the expression and function of these molecules for the dynamic regulation of mitochondria in long axon stretches are not well understood under physiological conditions or in complex diseases. Few key upstream modulating factors of the fission-fusion machinery have been identified (Anding et al., 2018, van der Bliek et al., 2013, Burman et al., 2017, Farmer et al., 2017, Otera and Mihara, 2011, Otera et al., 2013).

Oxidation and reduction (redox) reactions of the glutathione (GSH) pathway take place in all cells and are essential for vital metabolic processes, including the production of ATP. Glutathione S-transferases (GSTs), reductases (GR), and peroxidases (GPx) control redox homeostasis and tightly balance the ratio of the antioxidant GSH and the toxic species oxidized glutathione (GSSG) (Aquilano et al., 2014). Mitochondria can be affected by GSH redox changes that manifest in neurological diseases and stress response conditions by several mechanisms. First, mitochondria cannot produce GSH themselves, so they rely on its import from the cytoplasm for the adequate detoxification of reactive oxygen species (ROS) (Marí et al., 2009, Ribas et al., 2014). Second, GSSG accumulation can cause widespread oxidation of proteins and lipids, including essential mitochondrial proteins of the electron transport chain (Marí et al., 2009, Ribas et al., 2014). While large shifts in intracellular redox are detrimental to cells, small shifts in the GSH:GSSG ratio have been reported to induce mitochondrial-associated changes that are not associated with toxicity *in vitro* (Shutt et al., 2012).

In this study, we developed a high-throughput method to identify new molecules required for axonal mitochondrial maintenance *in vivo*. We discovered that a novel GST, Gfzf, homologous to GSTT1 in humans, regulates mitochondrial length in axons and functions by altering redox balance. Ablation of *gfzf* was found to cause the oligomerization of the outer mitochondrial membrane fusion factor Marf, a fly homolog of MFN, to initiate the fusion process and induce a complete fusion of the inner membrane through a secondary OPA1-mediated response. We show that mitochondrial fusion events also require the coordinated action of the specific GPx Jafrac1 and can be ameliorated by the expression of the GR TrxR-1. Loss of this GST caused significant functional changes within the neuron, including mitochondrial trafficking and electrophysiology. GSTs can therefore be classified as new essential upstream regulators of redox-mediated mitochondrial fusion.

## Results

### A High-Throughput *In Vivo* Genetic Screen for Modifiers of Axonal Mitochondria

**Figure 1:**
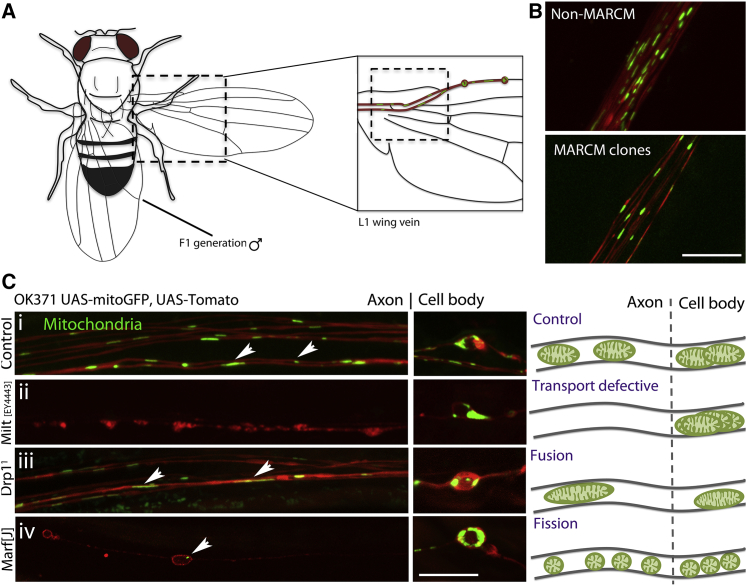
A Forward Genetic Screen to Analyze Mitochondria Dynamics in Axons (A) The mitochondria screen was designed such that male flies in the F1 generation were collected for wing dissection that was rapidly visualized by fluorescent microscopy at 7 days p.e. (B) Random mutations in the genome were induced by the chemical mutagen EMS, and the effect visualized in homozygous clones using the MARCM approach (mitochondria, green; neuronal clones, red). (C) The mitochondrial screen was verified using known mitochondrial modifying mutants of *Milton* [EY4443], *Drp1*^1^, and *Marf* [J], such that modifications in mitochondrial transport and length can be clearly observed at 7 days p.e. compared to controls in both the axonal and/or cell body cellular compartments, as depicted in the schematic. Arrows indicate significantly changed mitochondrial phenotypes in axons. Scale bars, 10 μm.

Mitochondrial maintenance processes present in non-polarized cells may occur in the axonal compartment of neurons (Amiri and Hollenbeck, 2008, Ashrafi and Schwarz, 2015, Ashrafi et al., 2014, Cartoni et al., 2016, Harbauer, 2017, Lee et al., 2018, Misgeld and Schwarz, 2017, Schwarz, 2013), but the similarities or differences remain poorly defined. We devised an *in vivo* forward genetic screen to identify new factors controlling mitochondrial dynamics in axons with single axon and mitochondrial resolution using *Drosophila* (Figure 1A). We used the MARCM system to visualize a subset of glutamatergic neurons in the adult *Drosophila* wing (Neukomm et al., 2014) and simultaneously labeled mitochondria (Figure 1B). Flies were fed the chemical mutagen ethyl methane sulfonate (EMS) and crossed to generate progeny containing MARCM clones in the F1 generation (Neukomm et al., 2017). These animals were screened for changes in mitochondrial number, size, and position. This approach allowed us to discover both lethal and nonlethal mutants that regulate mitochondrial in adult post-mitotic neurons *in vivo*.

To begin characterizing this system, we assayed the effects of loss-of-function manipulations of known molecules that regulate mitochondrial biology at 7 days post-eclosion (p.e.) (Figure 1C). Mitochondria in control neurons were distributed throughout the cell body and the axon and were stereotyped in size and density per unit axon area (Figure 1Ci). Induction of neuronal clones with a null mutation in the mitochondrial transport gene *Milton* resulted in the failure of mitochondria to enter the axonal compartment (Figure 1Cii), in agreement with Vagnoni et al. (2016). Mutations in mitochondrial fission gene *Drp1* caused fusion of mitochondria in the axon and a hyperfusion phenotype in the cell body (Figure 1Ciii; Figure S1). Mutants lacking the fusion factor Marf exhibited a significant shortening of mitochondria within the axon and a diffuse appearance in the soma (Figure 1Civ). Given the high efficiency and resolution of this system for examining mitochondrial phenotypes in axons in an *in vivo* setting and the potential to identify new regulators of mitochondrial physiology, we screened through ∼8,000 mutagenized chromosomes on the left arm of the 3^rd^ chromosome (representing ∼20% of the *Drosophila* genome). We discovered one lethal mutant, line #541, where mitochondria were significantly longer in axons compared to non-mutagenized controls, which we functionally characterize below.

### Loss of Gfzf Results in Increased Mitochondrial Length in Axons

A combination of whole-genome sequencing and deficiency mapping was used to identify a premature stop mutation in *GST-containing FLYWCH zinc-finger protein* (*gfzf*), a GST gene, that caused the long mitochondrial phenotype. Outcrossed flies that retained the phenotype were always associated with lethality. We found three overlapping deficiencies that failed to complement #541 in the genomic region of *gfzf* mutation revealed by the sequencing. Based on this observation and rescue experiments (below) we refer to mutant #541 as *gfzf*^−/−^.

**Figure 2:**
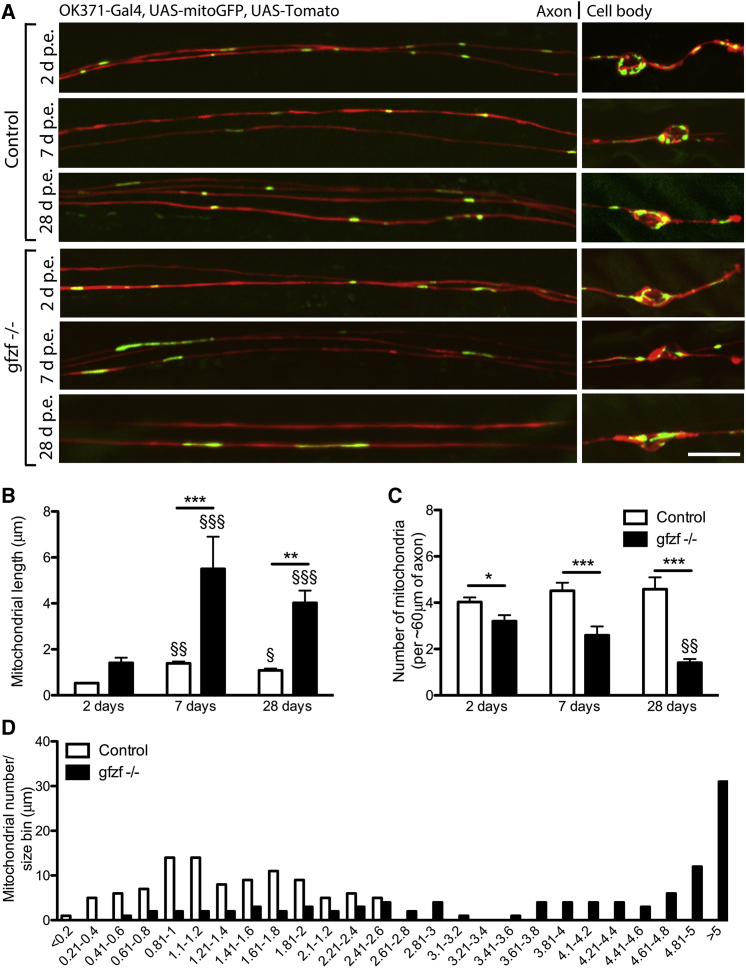
Mutations in a Novel Mitochondrial-Regulating Gene, *gfzf*, Cause an Increase in Mitochondrial Length (A) Mutations in the gene *gfzf* caused an age-dependent increase in mitochondrial length and a reduction in mitochondrial number, as visualized in the proximal region of the axons (mitochondria, green; neuronal clones, red). (B) The increase in mitochondrial length compared to control was not significantly different at 2 days p.e. and was increased 3- to 4-fold at both 7 and 28 days p.e. (C) Mitochondrial number was found to be marginally reduced at 2 days p.e. and greatly reduced in *gfzf* mutant axons at both 7 and 28 days p.e. (D) Analyzing the distribution of mitochondria lengths in the proximal axons at 7 days revealed that there was a significant shift in *gfzf*^−/−^ clones to lengths of ≥2.61 μm, with no control mitochondrial reaching these lengths. Data were analyzed by 2-way ANOVA and significant differences annotated as ^∗^p < 0.05, ^∗∗^p < 0.01, and ^∗∗∗^p < 0.001 between genotypes and ^§^p < 0.05, ^§§^p < 0.01, and ^§§§^p < 0.001 across age. Data in graphs are expressed as mean ± SEM and N ≥ 10 wings for each group. Scale bar, 10 μm.

Increased mitochondrial length in *gfzf*^−/−^ mutants was found to be age dependent (Figure 2). While only a minimal phenotypic difference was observed at 2 days p.e., excessively long mitochondria were observed at both 7 and 28 days (Figure 2A). Mitochondrial morphology was also altered in the neuronal cell bodies of *gfzf*^−/−^ mutant clones at later time points (Figure 2A). Quantification of mitochondrial length in axons revealed that at 7 and 28 days, mitochondria were on average 3–4 times longer than age-matched controls (Figure 2B), and the total number of mitochondria significantly decreased (Figure 2C). For instance, by 28 days p.e., one single long mitochondrion was observed in a 60 μm length of mutant axon, whereas control neurons contained ∼5 shorter mitochondria (Figure 2C). Analysis of the frequency distribution of mitochondrial length in *gfzf*^−/−^ mutant axons revealed a shift in length from a typical control range of 0.1–2.61 μm to a much broader range of 0.41 to >5 μm (Figure 2D).

In the experiments described above, mitochondrial lengths were analyzed in a defined region of the proximal wing (Figure 1A). However, given the substantial length of these glutamatergic neurons, we wished to determine whether the alterations in mitochondrial length observed in *gfzf*^#541^ mutants were present throughout the axon, and this was indeed the case (Figures S2A and S2B). A frequency distribution shift of mitochondrial length also manifested as animals aged (Figures S2C–S2E). Therefore, *gfzf*^−/−^ mutants affected mitochondrial length in axons independent of their location relative to the soma, and the effect increases with age. The total percentage axonal area occupied by mitochondria in the distal wing was further quantified within the axon and cell body compartments and not significantly changed (Figures S2F and S2G). This indicated that the mitochondrial phenotype is likely a fission-fusion deficit that does not affect biogenesis or degradation pathways.

**Figure 3:**
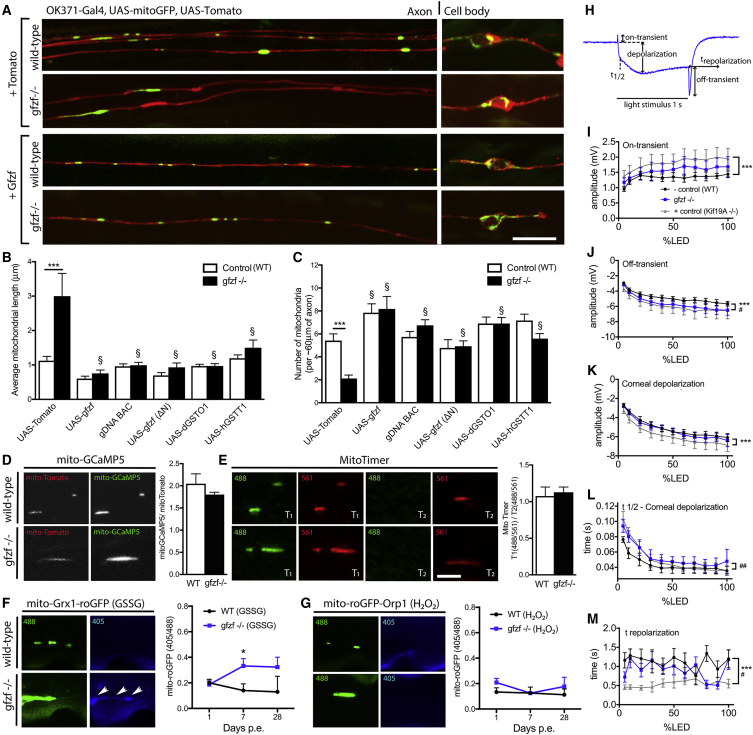
Increased Mitochondrial Length in *gfzf* Mutants Is Caused by GST Activity Altering Electrophysiological Properties (A) Increased mitochondrial length and reduced mitochondrial number were rescued to control levels by re-expression of the full-length cDNA of *gfzf*, as observed at 7 days p.e. (B) The increase in mitochondrial length compared to control was significantly rescued to control levels under the following conditions: expression of full-length *gfzf* cDNA, cDNA containing just the GST domain (ΔN), a genomic BAC duplication construct for *gfzf* expression, and cDNA for another GST-domain-containing gene, dGSTO1, and hGSTT1. (C) Decreased mitochondrial number also rescued to control levels under the same paradigms that were found to rescue mitochondrial length increases. (D–G) The function of the long mitochondria in *gfzf*^−/−^ clones was also assessed with *in vivo* live-cell imaging using mitochondrial-specific reporters for calcium (GCaMP5), age and maturation (mitoTimer), glutathione redox (Grx1-roGFP2), and H_2_O_2_ redox glutathione redox (roGFP2-Opr1). Mitochondria residing in mutant clones showed no significant functional changes in either calcium levels (D) or age (E), as measured by fluorescence intensity, at 7 days p.e. compared to control mitochondria. Reporters showed that GSSG levels were increased in mitochondria (F), yet H_2_O_2_ levels remained unchanged (G). (H) Electroretinogram (ERG) recordings were conducted in aged flies at 28 days p.e. using a GFP flipout clone system and the annotated parameters quantified. ERG was measured at a range of LED intensities. (I and J) The on-transient peak did not differ between WT and *gfzf*^−/−^ mutant backgrounds (I); however, significant changes in the off-transient peak were seen (J). (K–M) Corneal depolarization was not significantly changed (K); however, the half-time to corneal depolarization (L) and time to repolarization (M) were affected by *gfzf* ablation. A kinesin motor transport mutant, *Kif19A*^−/−^, was used as a positive control throughout. Data for rescue experiments were analyzed by 2-way ANOVA and significant differences annotated as ^∗∗∗^p < 0.001 between genotypes of the same treatment and ^§^p < 0.05 between baseline controls. Data for live-cell imaging experiments to determine mitochondrial function were analyzed by t test or 2-way ANOVA and significant differences annotated as ^∗^p < 0.05 between genotypes. For ERG, significant differences between *Kif19A*^−/−^ and control were annotated as ^∗∗∗^p < 0.001 and gfzf^−/−^ and control as ^#^p < 0.05, ^##^p < 0.01, and ^###^p < 0.001. Data in graphs are expressed as mean ± SEM and N ≥ 10 wings for each group or n = 6 flies (I–M) for each group. For roGFP experiments, 1–7 mitochondria were averaged per wing (depending on genotype). Scale bars represent 10 μm (top) and 4 μm (bottom).

To confirm that the stop mutation in *gfzf*^−/−^ was indeed responsible for the alterations in mitochondrial size, we expressed a full-length wild-type *gfzf* cDNA in *gfzf*^*#541*^ mutant clones. Upstream activating sequence (UAS)-mediated expression of *gfzf* was sufficient to rescue long mitochondrial phenotypes observed in the axons and cell bodies at 7 days p.e. (Figure 3A). Expression of *5xUAS-gfzf* significantly reduced mitochondrial length compared to baseline, equivalent to those observed in controls (Figures 3B and 3C). A genomic bacterial artificial chromosome (BAC) clone also fully rescued the mitochondrial phenotype in *gfzf*^*#541*^ mutants (Venken et al., 2009), (Figures 3B and 3C). Autonomous expression of *gfzfΔN*, which still harbors the GST domain (but lacks the zinc finger [ZNF] domains), was also sufficient to rescue the mitochondrial phenotype in *gfzf* mutants. These findings argue that the GST domain is the key regulatory domain in Gfzf necessary to modulate mitochondrial length in axons.

A range of other mutants of *gfzf* have been generated (Provost et al., 2006), outlined in (Figure S3A), but we found that the nature of these mutations was less severe than *gfzf*^*#541*^*. gfzf*^*#541*^ was the only allele that failed to complement the lethality of a deficiency lacking *gfzf*, or a transposon inserted in *gfzf Mi(MIC)gfzf*^*MI08697*^ (Figure S3B). We therefore propose that *gfzf*^*#541*^ is a null allele and will also refer to *gfzf*^*#541*^ as *gfzf*^−/−^. *gfzf*^*CZ811*^ also failed to complement most other alleles, including *gfzf*^−/−^ and the *Mi(MIC)gfzf*^*MI08697*^ line, and showed increased mitochondrial length and decreased mitochondrial number (Figures S3C–S3E). No clones were observed *gfzf*^*CL1027*^, suggesting this chromosome arm may harbor an additional cell lethal mutation. This independently derived allele further confirms that loss of Gfzf causes increased mitochondrial length. Rescue experiments indicate that embryonic lethality caused by Gfzf loss was likely not the result of neuronal-specific deficits, decreased GST activity, or mitochondrial fusion perturbations (Figure S3F). Therefore, Gfzf seems to have a dual role: an essential role in fly development in other tissues that does not depend on GST activity and a role to maintain mitochondrial length in adult neurons in which GST is both necessary and sufficient. The GST domain of Gfzf has a 30% identity and 48% homology with the GST domain of human GSTT1 (Figure S3G). Cas9 and guide RNA (gRNA)-mediated targeting of the mammalian GST homolog GSTT1 was also sufficient to increase mitochondrial length in cultured neurons *in vitro* (Figure S4), arguing for strong conservation of GST-mediated regulation of mitochondrial size.

### GST Regulates Mitochondrial Length and Neuronal Function

Mitochondrial phenotypes were first rescued by expression of Gfzf in *gfzf*^−/−^ clones confirming the causative gene (Figure 3A) before investigating the interaction with other GSTs. There are multiple GSTs in the *Drosophila* genome, all of which can likely regulate GSH:GSSG ratios. To determine whether the ability of the GST domain of Gfzf to regulate mitochondrial length was specific, we tested whether other cytoplasmic localized GSTs could compensate for Gfzf or directly regulate mitochondrial length in axons (Figures 3B and 3C). We found that the expression of *dGstO1* could reduce mitochondrial length in *gfzf*^−/−^ neurons (Figure 3B). This suggests that GST-domain-containing proteins in *Drosophila* have the general ability to regulate mitochondria and may function synergistically to determine axonal mitochondrial length. GST-domain-containing proteins are highly conserved between the fruit fly and humans. We further found that mitochondrial length in *gfzf*^−/−^ clones was rescued by expression of *hGSTT1*, (Figure 3B), but not *hGSTT2, hGSTO1*, *hGSTO2*, and *hGSTM1* (Table S1), arguing that human GSTT1 may be the most conserved GST-containing protein for maintaining mitochondria dynamics in neurons. Rescue of mitochondrial length was always accompanied with a rescue in number per unit of axon length (Figure 3C), supporting a causal link between these phenotypes.

Genetic manipulations that lead to dramatic changes in mitochondrial morphology are also tightly associated with perturbation of mitochondrial physiology and age-dependent neurodegeneration. We used *in vivo* live-cell imaging to assess mitochondrial Ca^2+^ levels, mitochondrial age and redox potential specific to GSSG and H_2_O_2_, in *gfzf* mutant clones, using *5xUAS*-*mito*-*GCaMP5, 5xUAS*-*mitoTimer*, *5xUAS*-*mito-Grx1-roGFP2* and *5xUAS*-*mito-roGFP2-Orp1* respectively (Figures 3D–3G). We detected no significant differences in the fluorescence intensity of mitochondrial localized *GCaMP5* (Figure 3D) and mitoTimer (Figure 3E). These data support the notion that mitochondria in *gfzf*^−/−^ mutant axons remain broadly functional. There was a significant increase in the fluorescence intensity depicting GSSG within the mitochondria from 7 days, which was not detectable in controls (Figure 3F). The fluorescence signal denoting H_2_O_2_ levels remained unchanged (Figure 3G). Electroretinogram (ERG) recordings in aged flies further indicated that these dynamic mitochondrial changes were associated with neuronal response phenotypes (Figures 3H–3M). Amplitude of the off-transient was reduced, the time to reach half-maximal corneal depolarization was delayed, and the time to fully repolarize was shortened in *gfzf*^−/−^ backgrounds relative to wild type (WT). Changes were rescued by a BAC clone or pan-neuronal knockdown of Marf, suggesting that physiological responses were directly related to the GSH pathway and mitochondrial hyperfusion (Figures S5A–S5C). *gfzf*^−/−^ mutant axons remain intact *in vivo*, and baseline Ca^2+^ levels were not significantly different from control, yet a significant decrease in mitochondrial transport was observed (Figures S5D–S5G). This indicates that large mitochondria had significantly higher GSSG content, which may impact bioenergetics, transport, and neuronal function.

### Cytoplasmic Gfzf Regulates Mitochondria in Multiple Neuronal Subtypes without Altering the Distribution of Other Axonal Organelles

We generated a transgenic stock containing *5xUAS-gfzf::GFP* and drove its expression using the pan-neuronal *synapsin1-Gal4* driver. Gfzf::GFP was observed throughout the neuron (Figure S6A). The localization of this molecule in cultured *Drosophila* S2 cells is also cytoplasmic (Dai et al., 2004). Regulation of mitochondrial size by Gfzf was also observed in other neuronal subtypes (Figures S6B–S6E). Interestingly, morphological changes in mitochondria were not observed at synaptic terminals or in glial clones (Figures S7A and S7B), suggesting that synaptic and glial mitochondrial size may be regulated by different mechanisms or other GSTs. Finally, we examined other organelles (lysosomes, endosomes, and peroxisomes) and observed no differences in distribution, number, and size of these vesicles between genotypes (Figures S7C–S7E). Together these data indicate that the Gfzf selectively regulates mitochondria in a pan-neuronal fashion and contributes to shaping the mitochondrial network in the axonal and cell body compartments.

### Increased Mitochondrial Length in *gfzf* Mutants Is Caused by Excessive Fusion

**Figure 4:**
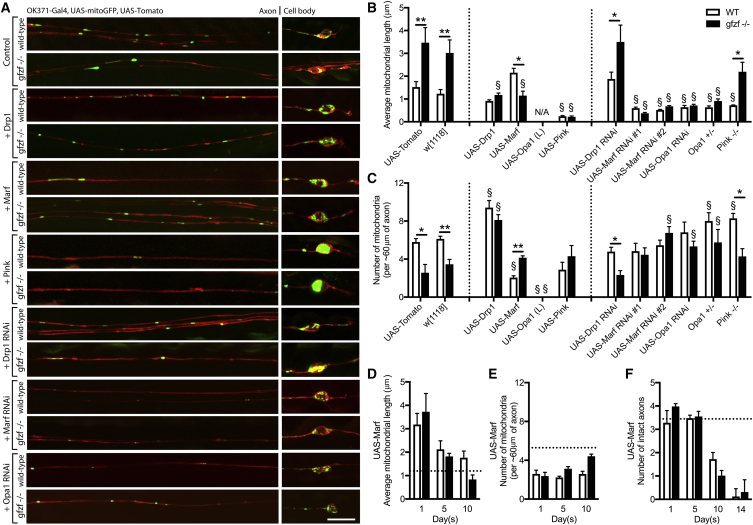
Increased Length in *gfzf*^−/−^ Mutant Clones Is the Result of Excessive Mitochondrial Fusion (A) Targeted epistasis experiments show how overexpressing and knocking down known mitochondrial regulating genes (fission, fusion and mitophagy) in control and *gfzf*^−/−^ mutant clones can alter mitochondrial distribution and size in the axon and cell body compartments. (B) Increased mitochondrial length phenotypes, observed at 7 days p.e., in *gfzf*^−/−^ mutant clones were rescued by OK371-Gal4-driven expression of *5xUAS-Drp1*, *5xUAS-Pink1*, *5xUAS-Marf*^*RNAi*^, and *5xUAS-Opa1*^*RNA*^ and in heterozygous *Opa1* mutants. Average mitochondrial length in control clones were increased above controls levels following expression of *5xUAS-Marf* in *gfzf*^−/−^ axons at 7 days. (C) Decreased numbers of mitochondria in the axon stretches of *gfzf*^−/−^ mutant clones were rescued by expression of *5xUAS-Drp1*, *5xUAS-Marf*^*RNAi*^, and *5xUAS-Opa1*^*RNAi*^ and in heterozygous *Opa1* mutants. *5xUAS-Marf* expression caused a reduction of mitochondrial number in control clones. (D) A biphasic shift in mitochondrial length was observed following expression *5xUAS-Marf* over time, causing a significant increase in mitochondrial length at 1 day p.e. in control clones, whereas long mitochondrial phenotypes seen in *gfzf*^−/−^ mutant clones remain unchanged compared to control levels (dashed line). At 5 and 10 days p.e., mitochondrial length was decreased, regardless of genotype. (E) Expression of UAS-Marf also causes a significant reduction in mitochondrial number at 1, 5, and 10 days p.e. compared to controls (dashed line), regardless of genotype. (F) Expression of *5xUAS-Marf* caused progressive neurodegeneration, and significantly fewer number axons were quantified at 10 and 14 days p.e. Data for rescue experiments were analyzed by 2-way ANOVA and significant differences annotated as ^∗^p < 0.05 and ^∗∗^p < 0.01 between genotypes following the same treatment and ^§^p < 0.05 and ^§§^p < 0.01 between experimental groups and their respective baseline controls. Data in graphs are expressed as mean ± SEM and N ≥ 10 wings for each group. Scale bar, 10 μm.

To explore the mechanism by which long mitochondria are generated in *gfzf* mutant clones, we examined the epistatic relationship between Gfzf and known fission and fusion factors. We found that increased expression of *Drp1*, *Marf*, or *Pink1* and RNAi-mediated knockdown of *Marf* or *Opa1* in adult neurons *in vivo* were sufficient to rescue the long mitochondrial phenotype of *gfzf*^−/−^ mutants (Figures 4A and 4B). Knockdown of the mitochondrial fusion factors *Opa1* and *Marf* caused a complete rescue of the long mitochondrial phenotype, which was also observed in double mutants of *gfzf*^−/−^, *Opa1*^*+/*−^ (Figure 4B). In each case, factors that increased mitochondrial length resulted in a decrease in mitochondrial number (Figure 4C). Interestingly, we found that promoting fusion by expression of Marf caused a biphasic shift in mitochondrial length and number (Figures 4D and 4E) and ultimately age-dependent neurodegeneration (Figure 4F). We simultaneously knocked down Marf in neurons overexpressing Gfzf, and no additive effect was observed, providing further epistatic evidence that Gfzf and Marf act in the same genetic pathway (Table S2). These observations indicate the increased mitochondrial length observed in *gfzf*^−/−^ mutant clones can be reversed by altering factors in the classical fission-fusion pathway and suggest that increased mitochondrial length in *gfzf* null clones is caused by hyperfusion of mitochondria. The GST activity of Gfzf is therefore a novel potential upstream modulator of inner and outer mitochondrial membrane fusion.

### Glutathione Redox Changes Regulate Mitochondrial Dynamics in Cultured Mouse Primary Neurons

**Figure 5:**
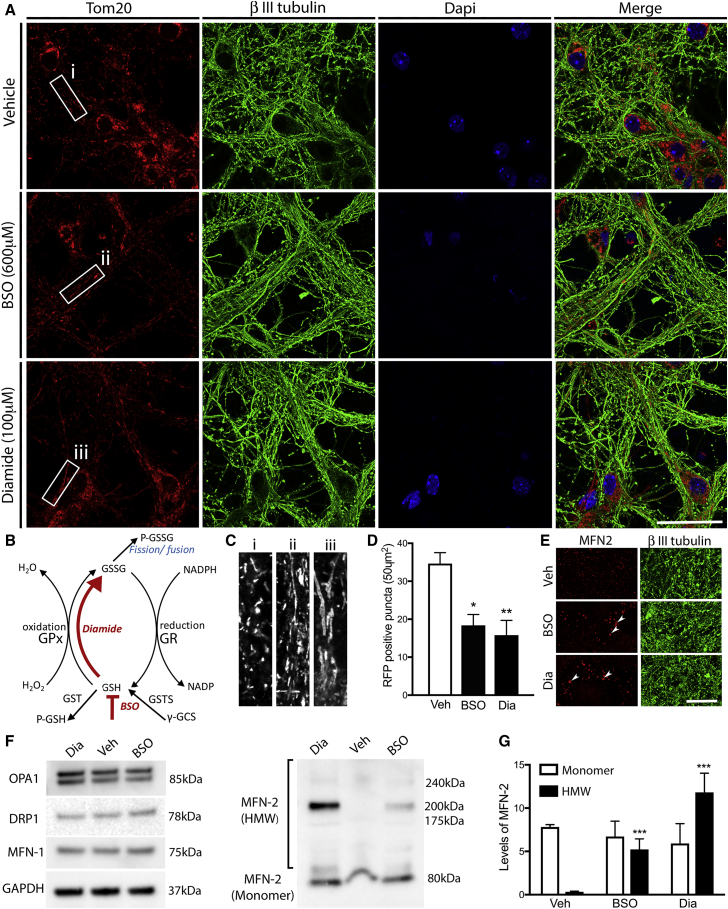
Pharmacologically Altering Glutathione Homeostasis in Mammalian Neurons *In Vitro* Results in Excessive Mitochondrial Fusion (A) 3-week-old primary cortical neurons were treated with either diamide (Dia) at a concentration of 100 μm for 2 h or L*-*buthionine-sulfoximine (BSO) at a concentration of 600 μm for 48 h. Following fixation, neurons and mitochondria were visualized using antibodies against ΒIII tubulin (green) and Tom20 (red), respectively, and the nuclei stained with DAPI (blue). (B) A schematic diagram illustrates the effect of Dia and BSO on the glutathione redox pathway. (C) High-magnification and contrast images show the change in mitochondrial morphology post-treatment. (D) A significant decrease in the number of Tom20-positive puncta was observed in axons following both Dia and BSO treatments. (E) The size of MFN2-positive puncta was also increased in axons following Dia and BSO treatments, as indicated by the arrows. (F) Biochemical analysis showed that levels of MFN1, OPA1, and DRP1 did not change compared to GAPDH following treatments but caused a specific increase in levels of MFN2 oligomers. (G) Oligomeric forms were significantly increased more than 10-fold following Dia treatment. Data were analyzed by 1-way ANOVA and significant differences annotated as ^∗^p < 0.05 and ^∗∗^p < 0.01 compared to vehicle treatment. Data in graphs are expressed as mean ± SEM and n = 3–6 for each group. Scale bars, 50 μm.

GSTs bind GSH to oxidized molecules and lipids for the purpose of cellular detoxification (Marí et al., 2009, Ribas et al., 2014), producing GSSG as a byproduct. We speculated that GSTs might modulate the capacity of Marf to drive mitochondrial fusion through a shift in the GSH:GSSG ratio. To test this possibility, we used two small-molecule treatments to change the redox state in cultured mouse primary cortical neurons: L*-*buthionine-sulfoximine (BSO) to block GSH synthesis (Dai et al., 2004) and diamide (Dia) to induce the rapid conversion of GSH to GSSG. We found that 48 h of BSO treatment or 1 h of Dia treatment resulted in clear morphological changes of mitochondria in cortical neuron projections (Figures 5A and 5B). Under these conditions, there was no evidence of cell death (Table S3). Mitochondria in neurites were significantly longer following BSO or Dia treatments compared to vehicle (Figure 5C), and the number of mitochondria was reduced compared to vehicle treatment (Figure 5D), phenocopying genetic *in vivo* data. These results show that reducing GSH:GSSG ratios initiates increases in mitochondrial length in mammalian neuronal projections.

To explore how increased GSSG might modulate known components of the mitochondrial fusion machinery, we examined mitofusin 2 (MFN2) localization in primary neuron cultures. Treatment with BSO or Dia resulted in MFN2-positive puncta that were larger in size and fewer in number compared to vehicle conditions (Figure 5E). This suggests that heightened GSSG levels might act by increasing oligomerization of MFN2 to promote fusion, as described previously (Shutt et al., 2012). Biochemical analysis further showed that the redox state had no obvious effect on fission-fusion factors other than MFN2 (Figures 5F and 5G). These experiments support the notion that GST-dependent modulation of glutathione redox balance is an evolutionarily conserved mechanism by which MFN2 oligomerization can be altered to modulate mitochondrial dynamics in axons.

### Mild Changes in GSH Redox Is Nondisruptive and Selective to Mitochondrial Fusion

**Figure 6:**
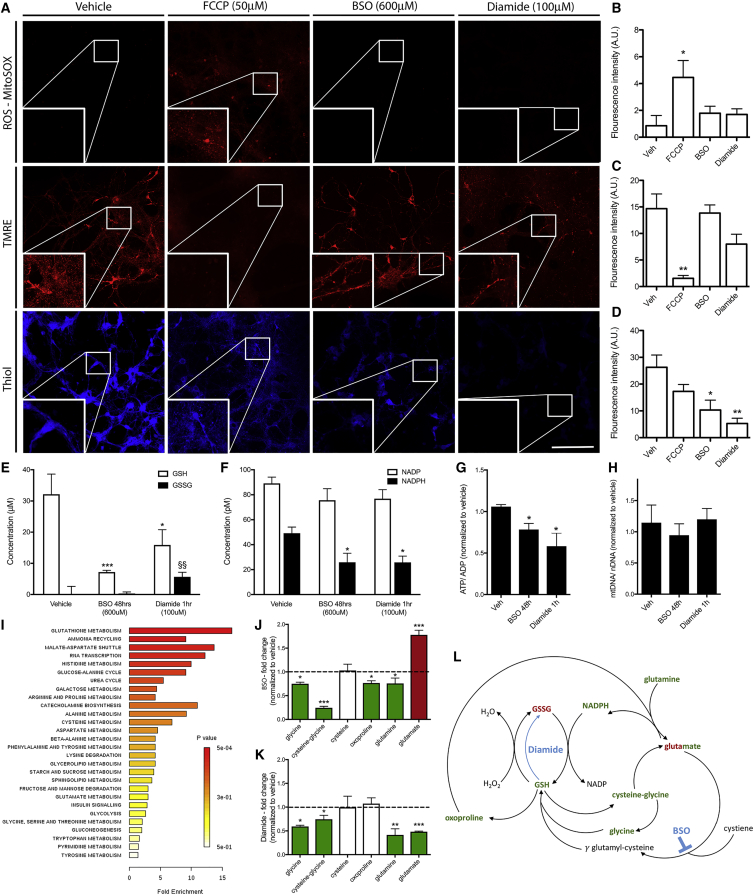
The Effect of Pharmacologically Induced Glutathione Imbalance on Mitochondrial Function and Metabolomic Profiles (A) Levels of reactive oxygen species (ROS) and mitochondrial membrane potential, determined by MitoSOX and TMRE, respectively, remained unchanged following Dia and BSO treatments for 2 and 48 h, respectively, but were significantly increased following treatment with FCCP (used as a positive control). Using a thiol-reactive dye, it could be observed that free thiols within neurons were reduced following both Dia and BSO treatments. (B and C) Quantification showed no effect of low dose Dia and BSO administration on ROS levels or mitochondrial membrane potential, as measured by fluorescence intensity of MitoSOX and TMRE. (D) Dia and BSO treatments caused a significant reduction in free thiol levels compared to vehicle treatment, as measured by fluorescence intensity and showed a marginal reduction following FCCP exposure. (E) Biochemical analysis of GSH and GSSG revealed a reduction of GSH following Dia and BSO administration. Dia treatment also caused a significant increase in GSSG levels. (F) Levels of the NADPH were also significantly decreased following both BSO and Dia treatments; however, this was not accompanied changes in NADP. (G) ATP/ADP ratios were also significantly reduced by the treatments. (H) Dia and BSO did not causes changes in mtDNA content. (I) Pathway analysis revealed that changes in the GSH pathway were the most enriched. (J) BSO treatment caused a significant decrease in the levels of glycine, cysteine-glycine, oxoproline, and glutamine and a significant increase in glutamate compared to vehicle (indicated by the dashed line). (K) Dia treatment caused a significant reduction of glycine, cysteine-glycine, glutamine, and glutamine. (L) Metabolomic changes were mapped onto to the KEGG GSH pathway and show that once GSH levels are reduced, either by blocking GSH synthesis (BSO) or by oxidizing GSH to GSSG (Dia), the balance cannot be recovered because of the lack of metabolic feedback. Data for immunofluorescence and mtDNA experiments were analyzed by 1-way ANOVA and significant differences annotated as ^∗^p < 0.05 and ^∗∗^p < 0.01 compared to vehicle. Data for GSH-GSSG and NADP-NADPH content were analyzed by 2-way ANOVA and significant differences annotated as ^∗^p < 0.05 and ^∗∗∗^p < 0.001 compared to vehicle and GSSG as ^§§^p < 0.01 compared to vehicle. Data for metabolite changes are expressed as fold change relative to vehicle, analyzed by 1-way ANOVA and annotated as ^∗^p < 0.05 compared to vehicle. Data in graphs are expressed as mean ± SEM and n = 4–6 for each group. Scale bar, 100 μm.

The dramatic changes in mitochondrial size we observed by manipulating GSTs *in vivo* or the GSH:GSSG balance *in vitro* might be predicted to have significant effects on mitochondrial metabolism or physiology. Similar to our *in vivo* findings, pharmacological treatments with BSO and Dia did not cause increased ROS or loss of mitochondrial membrane potential, visualized using live imaging with MitoSOX and tetramethylrhodamine ethyl ester (TMRE), respectively, compared to the carbonyl cyanide-4-(trifluoromethoxy)phenylhydrazone (FCCP)-treated positive control (Figures 6A–6C). However, levels of GSH, measured by ThiolTracker, were markedly reduced (Figures 6A and 6D). Biochemical analysis using cell lysates also showed that BSO and Dia treatment led to a reduction of GSH and, as expected, GSSG was significantly increased by Dia treatment, (Figure 6E). Normal homeostatic mechanisms would be predicted to allow for the conversion of GSSG back to GSH via NADPH. While NADP remained unchanged by BSO or Dia treatments, NADPH levels were significantly reduced (Figure 6F). Decreased NADPH levels were accompanied by a drop in the ATP:ADP ratio (Figure 6G), a relationship previously reported by monochlorobimane treatment (Vesce et al., 2005). BSO or Dia treatment did not have an effect on total mtDNA copy number, (Figure 6H), indicating that GSSG-regulated events did not enhance or decrease the total mitochondrial network size.

We next investigated the metabolic profile of neurons with alterations in GSH:GSSG ratios. A large number of metabolites were differentially altered following BSO and Dia treatment, and the GSH pathway was the most dysregulated by the GSH:GSSG ratio change, as expected (Figure 6I). BSO treatment also caused a significant decrease in glycine, cysteine-glycine, oxoproline, and glutamine (Figure 6J), and Dia treatment caused a similar change (Figure 6K). Other changes are annotated in Tables S4 and S5. Glutamate levels were drug dependent. Metabolite changes were mapped onto the mouse KEGG GSH pathway (Figure 6L), highlighting how changing GSH:GSSG ratios cause both a reduction in GSH conversion to oxoproline and synthesis of GSH as the result of lower glycine and cysteine levels (Figure 6L). We conclude that GST loss causes long-term changes in key metabolites of the GSH pathway, which can serve to ensure sustained hyperfusion through maintenance of the GSH:GSSG ratio imbalance.

### Gfzf Requires the Coordinated Action of the Thiol-Specific Peroxidase Jafrac1 and the Thioredoxin Reductase 1 TrxR-1

**Figure 7:**
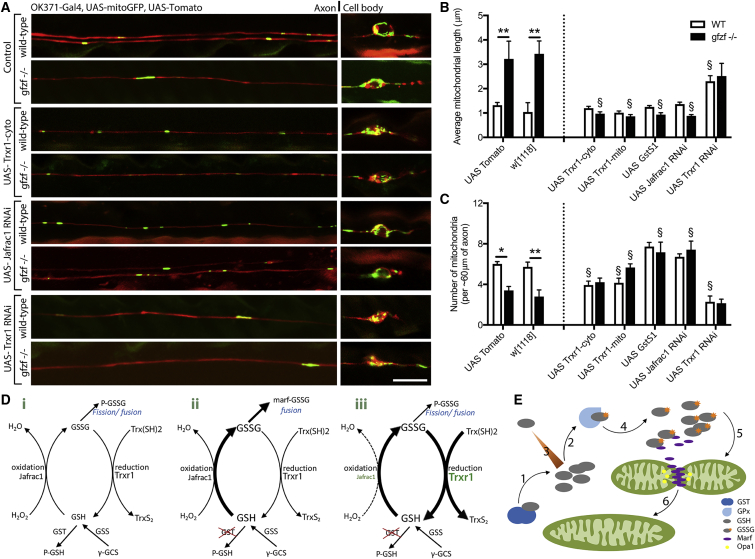
Gfzf Acts Synergistically with Jafrac1 and Trx1 for Fission-Fusion Balance (A) Targeted epistasis experiments show that long mitochondrial phenotypes caused by *gfzf* ablation in neuronal clones can be rescued by the pan-neuron expression of a reductase using *5xUAS-TrxR1-cyto* or knockdown of a specific oxidase using *5xUAS-Jafrac*^*RNAi*^. Mitochondria length in control neurons is enhanced by expression of *5xUAS-Jafrac1*^*RNAi*^. (B) Quantification shows that mitochondrial length was significantly reduced by *5xUAS-TrxR1-cyto*, *5xUAS-TrxR1-mito*, *5xUAS-GSTS1*, and *5xUAS-Jafrac1*^*RNAi*^, and mitochondrial length increased in control neurons by expression of *5xUAS-TrxR1*^*RNAi*^. (C) Mitochondrial number in *gfzf* mutant neuronal clones was significantly increased by *5xUAS-TrxR1-cyto*, *5xUAS-TrxR1-mito*, *5xUAS-GstS1*, and *5xUAS-Jafrac1*^*RNAi*^ expression, and mitochondrial number increased in control neurons by expression of *5xUAS-TrxR*^*RNAi*^. (D) A schematic diagram models how these molecules control normal glutathione homeostasis as depicted by reduced GSH/oxidized GSSG ratios (i). GST ablation was found to alter this balance in favor of increased mitochondrial fusion (ii); however, reducing levels GSSG by increasing levels of TrxR1 restores this imbalance (iii). (E) A model of how mitochondrial fusion can be induced based by ablation of GST and subsequent changes in glutathione metabolism. Following GST knockout, GSH is increased and rapidly (1) converted to GSSG by glutathione peroxidase (2). Metabolic changes cannot compensate of GSH depletion and the imbalance persists (3). GSSG accumulates (4) and causes the oligomerization of Marf in the axon (5) to induce the fusion of the inner and outer mitochondrial membranes (6). Data were analyzed by 2-way ANOVA and significant differences annotated as ^∗^p < 0.05 and ^∗∗^p < 0.01 between genotypes of the same treatment and ^§^p < 0.05 compared to genotype-matched baseline. Data in graphs are expressed as mean ± SEM and N ≥ 10 wings for each group. Scale bar, 10μm.

To further define how GSTs function to modulate the GSH:GSSG ratio and increase mitochondrial fusion in axons *in vivo*, we conducted targeted epistasis experiments, investigating key components of GSH synthesis and degradation pathway (Figure 7). In *Drosophila*, a typical NADPH-dependent GR is absent, and the observed GSH reduction is supported by the Trx-dependent GR TrxR-1 (Kanzok et al., 2001). Two alternatively spliced transcripts are transcribed from the *trxr-1* gene, which separately target either the mitochondria or the cytoplasm (Missirlis et al., 2002). Neuronal specific expression of either *TrxR-1-cyto* or *TrxR-1-mito* caused a reduction in excessive mitochondrial length and rescued the phenotype in *gfzf*^−/−^ clones (Figures 7A and 7B). This is consistent with the notion that the long mitochondrial phenotype is a direct consequence GSSG-induced Marf oligomerization in the cytoplasm and that either cytoplasmic or mitochondrial TrxR-1 can suppress *gfzf*^−/−^ mutant phenotypes. Increasing levels of GstS1 in *gfzf*^−/−^ mutants also rescued mitochondrial hyperfusion (Figures 7A–7C), likely by rebalancing GSH:GSSG ratios.

We next wanted to determine whether decreasing GSH oxidation through decreasing GPx activity was sufficient to impact mitochondrial size or modify *gfzf*^−/−^ mutant phenotypes. Jafrac1 is a major cytosolic peroxiredoxin and is the homolog of the mammalian *PRX2* gene (Orr et al., 2013). Silencing *Jafrac1* had no effect on mitochondria in control neuron clones, but it caused a complete rescue of mitochondrial phenotypes in a *gfzf* mutant background (Figures 7A–7C). These data support the notion that the decreased GSH levels that likely occur after GST deletion result from active conversion to GSSG in the cytoplasm via enzymatic oxidation. Finally, we also wanted to establish whether we could induce mitochondrial fusion by simply inhibiting the conversion of GSSG to GSH by eliminating the GR TrxR-1. Indeed, RNAi-mediated knockdown of *trxr-1* was sufficient to drive mitochondrial fusion in control neurons, phenocopying *gfzf*^−/−^ mutants, but it had no additive effect in *gfzf*^−/−^ mutant clones (Figures 7A–7C). These data are consistent with TrxR-1 and Gfzf acting in the same genetic pathway to modulate mitochondrial size.

Taken together, these results argue for a critical *in vivo* role for GSH:GSSG ratios in regulating mitochondrial morphology. Once GSSG levels are sufficiently high relative to GSH to induce excessive mitochondrial fusion in *gfzf*^−/−^ mutants, hyperfusion can be inhibited to normal levels again by increasing GSH synthesis or decreasing GSH oxidation. These data further indicate that inhibiting the enzymatic reduction of GSSG alone is sufficient to drive mitochondrial fusion *in vivo*.

## Discussion

The physiological role of mitochondrial remodeling remains underexplored, and the function of dynamic mitochondrial changes in neurons, specifically in neuronal sub-compartments, is even less understood (Eisner et al., 2018). We show that the GSH redox pathway controls the mitochondrial network in axons and have identified several molecules whose manipulation alters the size and shape of mitochondria *in vivo*. Specifically, we show that GSTs, known for their cellular detoxification roles in times of stress, also control the shape of the mitochondrial network, via fusion inhibition, under normal physiological conditions. Loss of a single GST-containing gene, *gfzf*, results in mitochondrial fusion *in vivo* through a GSSG-Marf interaction, impacting mitochondrial trafficking and neuronal response. This new role of GSTs provides a potential molecular explanation for how small molecules that change redox state can alter mitochondrial morphology *in vitro* (Shutt et al., 2012) through MFN oligomerization (Mattie et al., 2018).

We find that fission-fusion balance is maintained in adult post-mitotic neurons *in vivo* through the coordinated action of several components in the GSH redox pathway. Gfzf, TrxR-1, Jafrac1, and GstS1 (Figure 7Di), likely homologs of GSTT1, TXNRD2, PRX2, and GSTS1 in humans, respectively, tightly control GSH:GSSG ratios. Ablation of *gfzf* causes increased levels of GSSG through the conversion of GSH by Jafrac1 to promote fusion (Figure 7Dii). Excessive mitochondrial fusion can be rescued through overexpression of TrxR-1 and hence GSSG-GSH conversion (Figure 7D iii), which likely reduces levels of oxidized Marf. We propose that GST-containing proteins function upstream of Marf and Opa1 in the mitochondrial fusion pathway, as negative regulators, and that GSTs are key regulators of mitochondrial length in neurons *in vivo* (Figure 7E).

Although GSTs are considered the major ROS scavenging enzymes, we found that a knockout of a single GST gene *in vivo* or by modulating GSH:GSSG ratios *in vitro* using low doses of small molecules did not induce a detectable level of ROS. This suggests that small fluctuations in the GSH redox balance in favor of too much GSSG modulate mitochondrial dynamics with no overt adverse effects on mitochondrial function. Prolonged or larger increases of GSSG and/or decreases in GSH may increase effects of ROS and ultimately induce neurotoxicity. This highlights the sensitivity of axonal mitochondria to the fine balance between mitochondrial hyperfusion and cell death when the GSH redox pathway is dysregulated.

Our data show that cytoplasmically localized GSTs in the Omega and Theta classes can alter mitochondrial dynamics *in vivo*, and roles for GSTs are likely more complex and wide-reaching than previously thought. The presence of a GST domain within the Charcot-Marie-Tooth-linked gene *GDAP1*, perhaps acting in a similar way, may help explain previous results that show mitochondrial hyperfusion following knockdown (López Del Amo et al., 2015). This suggests that both classical GSTs and other GST-containing proteins may function cooperatively to modulate mitochondrial length.

Genome-wide association studies (GWAS) show that polymorphisms in GST genes (Allen et al., 2012) are associated with increased risk in developing Alzheimer’s disease (AD) and Parkinson’s disease (PD) later in life. Even in sporadic cases, redox state is significantly altered compared to age-matched controls (Chinta and Andersen, 2008, Saharan and Mandal, 2014, Sian et al., 1994, Smeyne and Smeyne, 2013). Polymorphisms may increase neuronal susceptibility during aging through both the accumulation of GSSG resulting in the subsequent oxidation of proteins and the long-term shortage of GSH needed to remove ROS. Consistent with this notion is the fact that expression of GSTs is upregulated in situations of heightened ROS in other cell types, including the closest homolog of *gfzf*, GSTT1 (Ito et al., 2011, Raza et al., 2002). It would be interesting to determine in future experiments whether there is an additive effect of oxidative stress using toxins in *gfzf* mutants that would make neurons more susceptible to degeneration.

GSSG-induced Marf oligomerization was sufficient to cause a complete fusion of mitochondria rather than solely the joining of mitochondria in axons to prime for their fusion later, since knocking down the inner mitochondrial membrane fusion protein Opa1 also resulted in a complete rescue of mitochondrial length. This indicates that outer mitochondrial membrane fusion is sufficient to trigger inner membrane fusion. Outer and inner mitochondrial fusion was previously found to be interdependent, since OPA1 cannot promote fusion in the absence of MFN (Cipolat et al., 2004). Fission-fusion dynamics have also been linked to deficits in mitochondrial transport (Chapman et al., 2013, De Vos et al., 2005, Niescier et al., 2016), with recent data showing that ROS levels can control mitochondrial motility through p38 and Miro and Trak (Debattisti et al., 2017). We find that GSSG-induced MFN-2 oligomerization caused significant mitochondrial trafficking problems. Therefore, movement deficits induced by a redox state change could be perturbed by two independent pathways occurring simultaneously.

In summary, we have demonstrated that GSTs are novel components of the mitochondrial fusion inhibition machinery *in vivo*. Our work provides a new link for a cellular pathway that is intimately involved with cellular responses to metabolic stress, and our study supports the notion that there are key differences in how different compartments of the neuron (i.e., cell body versus axon) regulate mitochondrial size *in vivo* in response to cellular metabolic changes. Future studies that explore the contributions of GST activity and changes in GSH:GSSG ratios in neurodegenerative disorders may provide important new mechanistic insights into how metabolic stress and changes in mitochondrial dynamics drive axonal loss in disease.

## STAR★Methods

### Key Resources Table

**Table:** 

REAGENT or RESOURCE	SOURCE	IDENTIFIER
**Antibodies**		

anti-beta III Tubulin	Abcam	Cat#ab107216; RRID: AB_10899689
anti-MFN2	Abcam	Cat#ab56889; RRID: AB_2142629
anti-Tom-20	ProteinTech	Cat#11802-1-AP; RRID: AB_2207530
488 goat anti-chicken	ThermoFisher	Cat#A32931; RRID: AB_2762843
Cy5 Goat anti-rabbit	Jackson ImmunoResearch	Cat#111-175-144; RRID: AB_2338013
Cy5 Goat anti-mouse	Jackson ImmunoResearch	Cat#115-175-146; RRID: AB_2338713
anti-Drp1	Abcam	Cat#ab56788; RRID: AB_941306
anti-Mitofusin 1	Abcam	Cat#ab57602; RRID: AB_2142624
anti-Mitofusin 2	Abcam	Cat#ab56889; RRID: AB_2142629
anti-Opa1	Abcam	Cat#ab42364; RRID: AB_944549
anti-GAPDH	Abcam	Cat#ab9485; RRID: AB_307275
HRP Goat anti-mouse	Abcam	Cat#ab6789; RRID: AB_955439
HRP Goat anti-rabbit	Abcam	Cat#ab6721; RRID: AB_955447

**Chemicals, Peptides, and Recombinant Proteins**		

Halocarbon Oil 27	Sigma	Cat#H8773
Ethyl methane sulfonate (EMS)	Sigma	Cat#M0880
Vectashield	Vector Laboratories	Cat#H1000
Trypsin-EDTA	ThermoFisher	Cat#25300054
DNase	Stem Cell Technologies	Cat#07900
MEM with L-glutamine	ThermoFisher	Cat#11095-080
Horse serum	ThermoFisher	Cat#26050070
Fetal Bovine Serum	Sigma	Cat#F9665
Neurobasal media	ThermoFisher	Cat#21103049
B-27	ThermoFisher	Cat#17504044
GlutaMax	ThermoFisher	Cat#35050061
Paraformaldehyde	EMS	Cat#15710
Dapi	ThermoFisher	Cat#D1306
SYBR PCR Premix	Takara Bio	Cat#639676

**Critical Commercial Assays**		

Glutathione Assay Kit	Cayman Chemical	Cat#703002
NADP/ NADPH colorimetric Kit	Abcam	Cat#ab186033
Bioluminescent ADP/ATP Ratio Assay Kit	Abcam	Cat#ab65313
MitoSOX Red	ThermoFisher	Cat#M36008
TMRE	Abcam	Cat#ab113852
Thiol-Tracker	ThermoFisher	Cat#T10095

**Experimental Models: Cell Lines**		

Primary embryonic neurons derived from time mated C57BL/6 mice	Charles River	https://www.criver.com/products-services/find-model/c57bl6-mouse?region=3671

**Experimental Models: Organisms/Strains**		

*OK371-Gal4*	BDSC	RRID: BDSC_26160
*GMR-myr::GFP*	BDSC	RRID: BDSC_7112
*Tubulin-Gal4*	BDSC	RRID: BDSC_5138
*Elav-Gal4*^*c155*^	BDSC	RRID: BDSC_458
n-Synaptobrevin-Gal4	BDSC	RRID: BDSC_51635
Nrv2-Gal4	BDSC	RRID: BDSC_6800
Cha-Gal4	BDSC	RRID: BDSC_6798
29A07-Gal4	Rubin Lab (Manning et al., 2012)	N/A
*10xUAS-IVS-myr::tdTomato*	BDSC	RRID: BDSC_32222
*5xUAS-mito::GFP*	BDSC	RRID: BDSC_8442
*FRT2A*	BDSC	RRID: BDSC_1997
*FRT82B*	BDSC	RRID: BDSC_2035
*tub-Gal80*	BDSC	RRID: BDSC_5135
ey-FLP	BDSC	RRID: BDSC_5577
*asense-FLP2c*	Freeman Lab (Neukomm et al., 2014)	N/A
*asense-FLP2e*	Freeman Lab (Neukomm et al., 2014)	N/A
*asense-FLP3b*	Freeman Lab (Neukomm et al., 2014)	N/A
*5xUAS-mito::tdTomato*	Freeman Lab (This paper)	N/A
*5xUAS-mCD8::GFP*	BDSC	RRID: BDSC_32192
*5xUAS-redStinger (NLS::Cherry)*	BDSC	RRID: BDSC_8546
*5xUAS-Drp1*	Feany Lab (DuBoff et al., 2012)	N/A
*5xUAS-TrxR1-mito*	Jackle Lab (Missirlis et al., 2002)	N/A
*5xUAS-TrxR1-cyto*	Jackle Lab (Missirlis et al., 2002)	N/A
*5xUAS-GstS1*	Pallanck Lab (Whitworth et al., 2005)	N/A
*5xUAS-lamp1::GFP*	BDSC	RRID: BDSC_42714
*5xUAS-Rab11::GFP*	BDSC	RRID: BDSC_8506
*5xUAS-SKL::GFP*	BDSC	RRID: BDSC_28880
*20xUAS-mito::GCaMP5*	Freeman Lab (This paper)	N/A
*20xUAS-GCaMP5*	Freeman Lab (This paper)	N/A
*5xUAS-roGFP2-Orp1*	BDSC	RRID: BDSC_67666
*5xUAS-Grx1-roGFP2*	BDSC	RRID: BDSC_67662
*UAS-mito-roGFP2-Orp1*	BDSC	RRID: BDSC_67667
*UAS-mito-Grx1-roGFP2*	BDSC	RRID: BDSC_67664
*5xUAS-MitoTimer*	BDSC	RRID: BDSC_57323
*5xUAS-Drp1*^*RNAi*^	BDSC	RRID: BDSC_51483
*5xUAS-Opa1*^*RNAi*^	VDRC	RRID: VDRCID_330266
*5xUAS-Marf*^*RNAi*^	BDSC	RRID: BDSC_55189
*5xUAS-Marf*^*RNAi*^	VDRC	RRID: VDRCID_105261
*5xUAS-Jafrac1*^*RNAi*^	VDRC	RRID: VDRCID_330046
Milton [EY4443]	BDSC	RRID: BDSC_19628
Drp1^1^	BDSC	RRID: BDSC_24885
Drp1^2^	BDSC	RRID: BDSC_24899
Marf [J]	BDSC	RRID: BDSC_57096
Opa1 [s3475]	BDSC	RRID: BDSC_12188
Pink1[5]	BDSC	RRID: BDSC_51649
Mi(MIC)*gfzf*^MI08697^	BDSC	RRID: BDSC_51102
*gfzf*^CZ811^	BDSC	RRID: BDSC_9436
*gfzf*^CU338^	BDSC	RRID: BDSC_9438
*gfzf*^DC806^	BDSC	RRID: BDSC_9439
*gfzf*^CL1027^	BDSC	RRID: BDSC_9437
*5xUAS-gfzf*^*RNAi*^	BDSC	RRID: VDRCID_25747
*5xUAS-gfzf*^*RNAi*^	BDSC	RRID: VDRCID_33932
*5xUAS-dGstO1*	Yim lab (Kim et al., 2012)	N/A
*5xUAS-hGSTO1-HA*	Bellen lab (This paper)	N/A
*5xUAS-hGSTO2-HA*	Bellen lab (This paper)	N/A
*5xUAS-hGSTT1-HA*	Bellen lab (This paper)	N/A
*5xUAS-hGSTT2-HA*	Bellen lab (This paper)	N/A
*5xUAS-hGSTM1-HA*	Bellen lab (This paper)	N/A
*5xUAS-gfzf*	Freeman lab (This paper)	N/A
*5xUAS-gfzf(ΔN)*	Freeman lab (This paper)	N/A
*5xUAS-gfzf::GFP*	Freeman lab (This paper)	N/A
BAC_CH322-97B15_ (genomic gfzf, landing site VK00037)	Freeman lab (This paper)	N/A
autosome deficiencies	BDSC	https://bdsc.indiana.edu/stocks/df/dfkit-info.htm

**Oligonucleotides**		

mtDNA primer 5′-CCC AAG CAT ATA AGC TAG TAC-3′	IDT	N/A
mtDNA primer 5′-ATA TAA GTC ATA TTT TGG GAA CTA C-3′	IDT	N/A
nDNA primer 5′-CGT GGG CTC CAG CAT TCT A-3′	IDT	N/A
nDNA primer 5′-TCA CCA GTC ATT TCT GCC TTT G-3′	IDT	N/A

**Software and Algorithms**		

Zen Blue	Ziess	https://www.zeiss.com
Zen Black	Ziess	https://www.zeiss.com
Prism7	Graph Pad	https://www.graphpad.com
ImageJ	Version 2.0	https://www.imagej.nih.gov
Slidebook 6	3i	https://www.intelligent-imaging.com/slidebook
MetaboAnalyst	Version 3.5	https://www.metaboanalyst.ca
ChromaTOF	Version 2.32	https://www.leco.com

**Other**		

### Contact for Reagent and Resource Sharing

Further information and requests for resources and reagents should be directed to and will be fulfilled by the Lead Contact, Gaynor Smith (Smithga@cardiff.ac.uk).

### Experimental Model and Subject Details

#### *Drosophila* Strains

All *Drosophila* strains (commercially available and generated) used are detailed in the Key Resources Table. The age and number used for each experiment are detailed in the figure legends. *Drosophila* strains used for mutagenesis and to generate MARCM clones: *OK371-Gal4, 10xUAS-IVS-myr::tdTomato, 5xUAS-mito::GFP, asense-FLP2c; FRT82B, tub-Gal80* (females) and *OK371-Gal4, 10xUAS-IVS-myr::tdTomato; FRT2A, FRT82B* (males). ey-FLP; FRT82B, *GMR-myr::GFP* was used for ERG experiments. The following *Drosophila* strains used for general experimental procedures and epistasis experiments: n-Synaptobrevin-Gal4, OK371-Gal4, Nrv2-Gal4, Cha-Gal4, R29A07-Gal4 (Rubin lab fly light collection), *FRT19A, FRT40A, FRT82B, asense-FLP2c, asense-FLP2e, asense-FLP3b, tub-Gal80, 10xUAS-IVS-myr::tdTomato, 5xUAS-mito::tdTomato, 5xUAS-mCD8::GFP, 5xUAS-mito::GFP, 5xUAS-redStinger (NLS::Cherry), 5xUAS-Drp1* (a gift from the Feany Lab (DuBoff et al., 2012)), *5xUAS-TrxR1-mito & 5xUAS-TrxR1-cyto* (a gift from the Langa and Jackle Labs (Missirlis et al., 2002)), *5xUAS-GstS1* (a gift from the Pallanck Lab (Whitworth et al., 2005)), *5xUAS-GstO1* (a gift from the Yim Lab (Kim et al., 2012), *5xUAS-lamp1::GFP, 5xUAS-Rab11::GFP, 5xUAS-SKL::GFP, 20xUAS-mito::GCaMP5, 20xUAS-GCaMP5, 5xUAS-roGFP-Orp1, 5xUAS-roGFP-Grx, UAS-mito-roGFP2-Orp1, UAS-mito-roGFP2-Grx* and *5xUAS-MitoTimer*. The following commercially available RNAi lines and mutants were used for further epistasis experiments and as positive controls for the mutagenesis screen: *5xUAS-Drp1*^*RNAi*^ *(51483), 5xUAS-Opa1*^*RNAi*^ *(330266), 5xUAS-Marf*^*RNAi*^ *(55189), 5xUAS-Marf*^*RNAi*^ *(105261), 5xUAS-Jafrac1*^*RNAi*^ *(330046)*, *Milton* [EY4443], *Drp1*^1^, *Drp1*^2^, *Marf* [J], *Opa1* [s3475] and Pink1[5]. Other commercially available gfzf alleles, deficiency lines spanning the region and RNAi lines tested were: Mi(MIC)*gfzf*^MI08697^, *gfzf*^CZ811^, *gfzf*^CU338^, *gfzf*^DC806^, *gfzf*^CL1027^, Df BSC221, *5xUAS-gfzf*^*RNAi*^ (25747) and *5xUAS-gfzf*^*RNAi*^ (33931).

A mixture of male and female flies were used throughout. No sex specific differences were observed. The majority of experiments were conducted at 7 days post eclosion (p.e.) with time course experiments done at 1, 7 and 28 days p.e. Flies which showed clear physical damage to the wings were not selected.

#### Generation of *Drosophila* lines

The plasmids listed below were generated by standard sub-cloning procedures, using a *5xUAS, w*^*+*^ marker backbone and injected into embryos using Phi31 integration by BestGene: *5xUAS-gfzf, 5xUAS-gfzf(ΔN), 5xUAS-gfzf::GFP*. *Drosophila* harboring the BAC_CH322-97B15_ were made through recombineering by BestGene. Gateway cloning was used to generate the following human cDNA constructs in pUASg-HA.attB (Bischof et al., 2013), used for rescue experiments: *UAS-hGSTO1-HA, UAS-hGSTO2-HA, UAS-hGSTT1-HA, UAS-hGSTT2-HA and UAS-hGSTM1-HA* (human constructs and transgenic stocks made in the Bellen Lab at Baylor College of Medicine).

#### Primary Culture

Cortical neuronal cultures were prepared from dissected embryos at E15, using wild-type naive time pregnant mice of the C57BL/6 strain (Charles River) and left to mature for 2 weeks before experiments conducted. Time pregnant mice were housed overnight in standard conditions and anaesthetized using isoflurane. Procedures were carried out according to the Institutional Animal Care Committee at the Vollum Institute (Oregon Health and Sciences University). A mixture of male and female embryos were used. Whole cortices were removed in HBSS-HEPES buffer, washed 10x and incubated in Trypsin/DNase for 5 mins at 37°C. Tissue was washed 3x before mechanical dissociation using standard pipetting procedure. Cells were centrifuged at 800rpm for 5 mins and resuspended into a single cell solution in MEM with L-glutamine, 10% Horse serum and 10% Fetal Bovine Serum to a density of 0.5 × 10^6^. Cells were plated onto Poly-L-lysine coated coverslips or plates and incubated at 37°C for 24 h. Media was then replaced with Neurobasal media containing B-27 and Glutamax, replenished every 2-3 days for 2 weeks.

### Method Details

#### Mutagenesis Screen for mitochondrial modifying phenotypes

Axonal MARCM clones were induced according to (Neukomm et al., 2014), and residing mitochondria were visualized in the L1 vein of each wing using *UAS-mito::GFP*. Any F1 male flies that displayed mitochondrial phenotypes were saved, bred, and outcrossed, as defined in (Neukomm et al., 2017). *Drosophila* stocks were kept on standard cornmeal and agar supplemented with dry yeast at 25°C. The mutagenesis screen was carried out using males that were starved for 8 h before consuming mutagen for a further 12 h (25mM ethyl methane sulphonate (EMS) in 1% sucrose). Males were recovered in fresh vials for 12 hours before breeding. The right wings of anesthetized F1 flies, aged 7 days at 25°C, were cut as close to the body as possible, using dissection Scissors (EMS, VANNAS). 20-30 wings were then mounted in Halocarbon Oil 27 (Sigma) on a microscopy slide, a coverslip placed on top, and then used immediately for microscopy. Flies were returned to vials in a numerical order relative to order of the wings.

#### *In vivo* live cell imaging

Live cell imaging was carried out according to a modified protocol by (Vagnoni and Bullock, 2016). Following CO_2_ anesthesia, the head, thorax and legs of the fly were additionally restrained by low melt agarose (Sigma), which was poured into a 5 mm indentation hollowed from a glass slide. A glass coverslip was placed directly on top of the wings held perpendicular to the body by Halocarbon Oil 27 (Sigma) and used immediately for imaging. 10-minute interval videos were used for each fly and image acquisition was carried out every 2 s. 4 animals were used per group and videos analyzed in a blind randomized fashion.

#### Electrophysiology

ERGs from flies were recorded as described previously (Fabian-Fine et al., 2003) and (Bayat et al., 2012), with Ag/AgCl wire electrodes sheathed with glass micropipettes containing Beadle-Ephrussi ringer. A fluorescence excitation light source (CoolLed pE-2 fitted with a 470 nm LED attenuated through a neutral density filter) was used to provide 1 s pulses of light. LED output was regulated to provide different stimulus intensities, with 50% output corresponding approximately to 0.2 mW/cm^2^ as converted from illuminance measurements using a light meter (Model 230, CHY-Firemate, Taiwan). Flies were allowed to adapt for 5 min to ambient light (3 lux) before experiments. 6 animals were used per group and groups were blind to blind to the investigator. Signals were digitized at a 1 kHz rate and analysis of the response variables was automated using a custom script in Igor Pro.

#### Immunocytochemistry

Primary neuronal culture medium was removed from cells adhered to coverslips and were washed with PBS (with Ca^2+^ and Mg^2+^ and fixed using 4% PFA at room temperature for 15 min. Neurons were washed 3x in PBS, permeabilized in PTX for 5 min and washed 3x in PBS. Cells were incubated in 5% horse serum in PBS for 60mins and incubated in the blocking solution with the following primary antibodies at 4°C overnight: Chicken anti-beta III Tubulin (ab107216) was used at a concentration of 1:1000, mouse anti-MFN2 (ab56889) used at 1:500 and rabbit anti Tom-20 (11802-1-AP) used at 1:250. Primary neurons were washed 5x in PBS and further incubated for 2 h at room temperature with: 488 goat anti-chicken (Jackson lab) and Cy5 Goat anti-rabbit/ anti-mouse (Jackson lab). Coverslips were washed 3x PBS, stained with Dapi (Fisher), washed 5x PBS and mounted in Vectashield. Experiments were repeated 6 times, each run in triplicate and 3 images averaged per well and mitochondria analyzed in a blind automated fashion.

#### Biochemistry

Western Blot analysis was performed on neuronal cell lysates following pharmacological treatments with BSO and Diamide. Protein levels were normalized using a Bradford assay and resolved by SDS–PAGE, transferred to polyvinylidene difluoride membrane and probed with the following antibodies: Drp1 (Abcam ab56788), Mitofusin 1 (Abcam ab57602), Mitofusin 2 (Abcam ab56889), Opa1 (Abcam ab42364) and GAPDH (Abcam ab9485). Bands were visualized using Hrp conjugated antibodies (Abcam). 3 samples were used per group and experiments were run in triplicate.

#### Drug treatments

Two week old primary cultures were treated with either diamide (Dia) at a concentration of 100μm for 2hours or L*-*Buthionine-sulfoximine (BSO) at a concentration of 600μm for 48hours, following an initial dose response curve to determine a dose that does not cause toxicity. Drugs were dissolved in DMSO

#### GST KO cell generation

One week old primary cultures were transfected with gRNA/Cas9 Knockout (KO) plasmids using sequences targeting mouse GSTT1 (SC-420721) or were scrambled (SC-418922), using GeneJuice (70967 Merck) according to the manufacturer’s instructions. Cells were left in culture for 1 week and selected by the GFP marker for further analysis. Experiments were repeated 4 times, each run in triplicate and 3 cells analyzed per well.

#### Analysis of mtDNA content

mtDNA was quantified using real-time (RT) qPCR, performed according to (Fuke et al., 2011), using SYBR Premix Ex Taq reagent (Takara Bio, Otsu, Japan) according to the manufacturer’s instructions. For estimation of amounts of mtDNA in mouse primary cortical neurons, mtDNA was amplified using the primer pair (5′-CCC AAG CAT ATA AGC TAG TAC-3′) and (5′-ATA TAA GTC ATA TTT TGG GAA CTA C-3′), used in the following qRT-PCR protocol: 95°C for 20 s, 55°C for 20 s, 72°C for 80 s (30 cycles). To quantify nDNA, the apoB gene was used for reference. Amplification was carried out using the following primer pair (5′-CGT GGG CTC CAG CAT TCT A-3′) and (5′-TCA CCA GTC ATT TCT GCC TTT G-3′) using the following protocol: 40 cycles of two-step PCR prior to: 95°C for 1 min, 95°C for 10 s and 60°C for 30 s. These real-time qPCRs were carried out in quadruplicate.

#### GSH/ GSSG, NADP/ NADPH & ATP/ADP biochemical analysis

For GSH/GSSG quantification primary cortical neurons were washed in PBS, dissociated and pelleted by centrifugation. Cells were then homogenized, the supernatant extracted, deprotienated and used immediately in a 96 well assay format. Quantitative detection of GSH and GSSG was carried out using a Glutathione Assay Kit (Cayman Chemical) according to the manufacturer’s instructions. Standards and experimental samples were run in duplicate on a microplate reader (SpectraMax) set to measure absorbance at 405nM. For detection of NADP/NADPH primary neuronal cultures were extracted and prepared by buffers provided by the NADP/ NADPH colorimetric Kit (Abcam). Detection was measured, 2 h after final incubation step, using the microplate reader set at 450nM. ATP/ADP ratios were analyzed using the Bioluminescent ADP/ATP Ratio Assay Kit (ab65313) according to the manufacturer’s instructions. Cell lysate samples were also run in triplicate and experiments repeated 4-6 times.

#### LDH release assay for cell death

Neurons were seeded in 96 well plates and were left to mature for 2 weeks Following appropriate drug treatments cell death was analyzed according to (Smith et al., 2016), using an LDH release assay kit (Cayman Chemical). N = 3 per group. Controls and experimental wells were run in triplicate.

#### Live fluorescent dyes

For live cell imaging experiments primary neurons were adhered directly to the bottom of Poly-L-Lysine coated 12-well plates. For the analysis of mitochondrial derived mitochondrial derived reactive oxygen species (ROS), mitochondrial membrane potential and free thiols in live primary neuronal mouse cortical cultures following drug treatments, MitoSOX (Fisher), TMRE (Abcam) and Thiol-Tracker (Fisher). Live fluorescent probes were used according to the manufacturer’s instructions, including doses and times stated. Optimized methods for this experiment: TMRE - 100nM for 20mins incubation. Experiments were repeated 4-6 times and each sample run in triplicate. 3 images were taken per well and averaged. Images were analyzed in an investigator blind fashion.

#### Whole-Genome Sequencing

Gene mutations responsible for mitochondrial phenotypes were discovered through the application of next generation sequencing. gDNA was extracted from ∼200 heterozygous male mutant adult flies and directly sequenced on a HiSeq2000 next-generation sequencing platform (Illumina). Bioinformatics analysis for read alignment and variant investigation was carried out through at the University of Miami Miller School of Medicine, Center for Genome Technology.

### Quantification and Statistical Analysis

#### Microscopy

For the visualization of mitochondrial and axons, in unfixed wings or fixed brains were imaged on a 3I spinning disc confocal microscope or on a Zeiss LSM 880 microscope. Data acquisition was carried out using Slidebook 6 or Zen Blue and Zen black software packages.

#### Metabolomics

Metabolomic analysis was carried out at the University of California Davis Metabolomics Core, using a Leco Pegasus IV mass spectrometer. ChromaTOF v2.32 was used for data preprocessing. Methodological details to be found in (Fiehn et al.). MetaboAnalyst v3.5 was used for statistical and pathway enrichment analysis and changes referenced to the KEGG pathway: glutathione metabolism - *Mus musculus* (mouse).

#### Analysis

Image processing was carried out with either Slidebook 6, Zen blue or Zen black and ImageJ 2.0 used for quantification of: mitochondrial length, mitochondrial number, fluorescence intensity and counting of puncta. All statistical tests were carried out using Prism 7 unless otherwise stated. Information on statistical tests used for each experiment and number of samples is indicated in the Figure legends. Statistical methods were chosen to fit data distributions and the number of groups per experiment as determined using Prism 7. For *in vivo* experiments where mitochondrial length and number were quantified at least 10 animals were quantified per group, 1-7 axons quantified for each animal. For *in vitro* experiments at least 3 separate experiments were performed, each run in triplicate.

## Supplemental Information

Document S1. Tables S1–S5 and Figures S1–S7

Document S2. Article plus Supplemental Information
