# Supplementary material for: Glutathione S-Transferase Regulates Mitochondrial Populations in Axons through Increased Glutathione Oxidation
Source: Neuron. 2019 Jul 3;103(1):52–65.e6. doi: 10.1016/j.neuron.2019.04.017 (PMC6616599; doi:10.1016/j.neuron.2019.04.017)
Supplement: Document S1. Tables S1–S5 and Figures S1–S7 [file mmc1.pdf]

Neuron, Volume *103*

Supplemental Information

Glutathione S-Transferase Regulates Mitochondrial Populations in Axons through Increased Glutathione Oxidation

Gaynor A. Smith, Tzu-Huai Lin, Amy E. Sheehan, Wynand Van der Goes van Naters, Lukas J. Neukomm, Hillary K. Graves, Dana M. Bis-Brewer, Stephan Züchner, and Marc R. Freeman

Supplementary Material

Glutathione-S-transferase regulates mitochondrial populations in axons through increased glutathione oxidation

Smith G.A.[1,2,3]*, Lin T-H. [1,2]*, Sheehan A.E.[1,2], Van der Goes van Naters W.[4], Neukomm L.J.[1,5], Graves H.K.[6], Bis-Brewer D.M.[7,8], Züchner S.[7,8], Freeman M.R[1,2]

[1]Department of Neurobiology, University of Massachusetts Medical School, Worcester, MA 01605, USA
[2]Vollum Institute, Oregon Health & Science University, Portland, OR, 97239, USA
[3]Dementia Research Institute, School of Medicine, Cardiff University, Cardiff, CF24 4HQ, UK.
[4]Molecular Biosciences, School of Biosciences, Cardiff University, Cardiff, CF10 3AX, UK.
[5]Department of Fundamental Neurosciences, University of Lausanne, 1005 Lausanne VD, Switzerland
[6]Department of Molecular and Human Genetics, Baylor College of Medicine, Houston, TX 77030, USA
[7]John P. Hussman Institute for Human Genomics, University of Miami, Miami, FL, USA.
[8]Dr. John T. Macdonald Foundation Department of Human Genetics, University of Miami, Miami, FL, USA

*Authors have equal contribution

Correspondence:

Gaynor A. Smith
Dementia Research Institute
School of Medicine
Cardiff University
Cardiff, CF24 4HQ, UK
Phone: (+44) 2922 510897
Email: SmithGA@cf.ac.uk

Supplementary Table 1. Rescue of mitochondrial phenotypes was specific to human GSTT1. Related to Figure 3.

Construct	Quantification type	WT	SEM	P[1]	gfzf-/-	SEM	P[1]
5xUAS-hGSTO1	Mitochondrial length	1.164	0.126	NS	2.777	0.521	NS
5xUAS-hGSTO2	Mitochondrial length	1.042	0.087	NS	2.704	0.297	NS
5xUAS-hGSTT2	Mitochondrial length	1.211	0.032	NS	2.833	0.491	NS
5xUAS-hGSTM1	Mitochondrial length	1.013	0.066	NS	3.219	0.630	NS
5xUAS-hGSTO1	Mitochondrial number	8.666	0.571	NS	4.818	0.383	NS
5xUAS-hGSTO2	Mitochondrial number	8.000	0.508	NS	3.000	0.516	NS
5xUAS-hGSTT2	Mitochondrial number	8.666	0.202	NS	2.363	0.312	NS
5xUAS-hGSTM1	Mitochondrial number	6.250	0.440	NS	2.909	0.706	NS

[1]Statistical analysis was carried out by 2-way ANOVA and p-value annotated as difference compared to *5xUAS-Tomato* expressed in the same genetic background

Supplementary Table 2. Non-significant metabolic changes assocated with increased oxidized glutathione. Related to Figure 4.

Construct	Mitochondrial measurement	Mean[1]	SEM	P[2]
5xUAS-gfzf	length	6.891	0.4112	--
5xUAS-Marf RNAi	length	7.002	0.3976	NS
5xUAS-gfzf & 5xUAS-Marf RNAi	length	5.566	0.664	NS
5xUAS-gfzf	number	7.861	0.766	--
5xUAS-Marf RNAi	number	4.132	0.300	<0.05
5xUAS-gfzf & 5xUAS-Marf RNAi	number	4.375	0.419	<0.05

[1]Mean expressed as a fraction of control *10xUAS-Tomato*.
[2]Statistical analysis was carried out by 1-way ANOVA and p-value annotated as difference compared to *5xUAS-gfzf*.

Supplementary Table 3. Neuronal viability following application of glutathione level depleting drugs. Related to Figure 5.

Drug	Application time	Dose (µM)	% cell death[1]	SEM
BSO	24 hr	50	0.027	0.027
		100	0.001	0.000
		200	0.031	0.030
		600	0.357	0.313
		1000	0.412	0.403
		2000	30.744	6.810
		lysed control	69.246	9.971
BSO	48 hr	50	0.0373	0.030
		100	0.003	0.001
		200	0.478	0.257
		600	1.239	0.241
		1000	37.883	11.485
		2000	75.343	8.067
		lysed control	81.600	9.599
		50	0.044	0.039
Diamide	1 hr	100	0.428	0.427
		200	5.694	2.510
		600	47.390	3.822
		1000	65.744	7.203
		2000	70.722	18.657
		lysed control	82.846	9.183
Diamide	2 hr	50	3.024	1.162
		100	15.509	8.376
		200	41.127	4.933
		600	77.815	6.549
		1000	80.519	7.634
		2000	75.399	15.917
		lysed control	79.076	1.904

[1] % LDH release compared to vehicle

Supplementary Table 4. Significant metabolic changes associated with increased oxidized glutathione. Related to Figure 6.

BinBase name	PubChem	KEGG	Fold change BSO V vehicle	SEM	P	Fold change Dia V vehicle	SEM	P
1-monostearin	24699	D01947	0.653	0.142	p<0.05	0.989	0.150	NS
2,3-dihydroxypyridine	28115		0.653	0.224	p<0.05	0.482	0.173	p<0.05
2,5-dihydroxypyrazine NIST	23368901		0.070	0.008	p<0.05	1.492	0.303	NS
3-phosphoglycerate	724	C00597	1.503	0.130	p<0.05	2.138	0.304	p<0.05
4-hydroxybutyric acid	10413	C00989	1.424	0.165	p<0.05	2.166	0.707	p<0.05
adenosine	60961	C00212	0.511	0.057	p<0.05	0.382	0.018	p<0.05
adenosine-5-monophosphate	6083	C00020	0.530	0.047	p<0.05	0.369	0.041	p<0.05
aspartic acid	5960	C00049	1.684	0.059	p<0.05	0.847	0.103	NS
butyrolactam NIST	12025		0.956	0.074	NS	0.358	0.129	p<0.05
creatinine	588	C00791	0.631	0.041	p<0.05	0.645	0.068	NS
D-erythro-sphingosine	5280335	C00319	1.223	0.047	NS	0.737	0.094	p<0.05
erythritol	222285	C00503	0.927	0.025	NS	2.912	0.495	p<0.05
fructose	439709	C02336	0.768	p<0.05	NS	1.259	0.237	NS
gluconic acid	6857417	C00800	0.634	0.083	p<0.05	3.844	0.226	p<0.05
glucose	64689	C00221	0.664	0.056	p<0.05	1.115	0.128	NS
glucose-1-phosphate	65533	C00103	1.322	0.585	p<0.05	1.144	0.738	NS
glyceric acid	439194	C00258	1.914	0.097	NS	3.529	0.120	p<0.05
glycerol-3-galactoside	16048618	C05401	1.219	0.116	p<0.05	1.423	0.255	NS
guanosine	6802	C00387	1.171	0.271	NS	0.577	0.152	p<0.05
hexadecylglycerol NIST	72733	C13859	0.583	0.112	p<0.05	0.960	0.198	NS
inosine	6021	C00294	1.315	0.092	p<0.05	0.862	0.165	NS
isothreonic acid	151152	C00639	0.969	0.082	NS	4.784	0.441	p<0.05
lactic acid	612	C01432	0.936	0.136	p<0.05	1.127	0.130	NS
lactobionic acid	7314	C04247	1.336	0.186	p<0.05	1.590	0.408	NS
lactulose	11333	C07064	1.158	0.166	NS	1.690	0.322	p<0.05
malic acid	525	C00711	1.018	0.007	NS	0.600	0.027	p<0.05
myo-inositol	892	C00137	0.740	0.035	p<0.05	0.739	0.037	NS
n-acetyl-d-hexosamine	24139	C03878	1.076	0.112	NS	10.198	0.699	p<0.05
N-acetylaspartic acid	65065	C01042	0.806	0.033	p<0.05	0.816	0.055	p<0.05
O-phosphoserine	57689797	C01005	0.640	0.067	p<0.05	1.108	0.301	NS
octadecanol	8221		0.647	0.090	p<0.05	1.132	0.287	NS
orotic acid	967	C00295	1.310	0.080	p<0.05	1.217	0.185	p<0.05
oxalic acid	971	C00209	0.997	0.180	NS	1.692	0.168	p<0.05
palatinitol	88735		0.751	0.116	p<0.05	1.093	0.129	NS
palmitoleic acid	445638	C08362	0.908	0.234	NS	1.372	0.077	p<0.05
pantothenic acid	6613	C12276	0.749	0.042	p<0.05	0.784	0.191	p<0.05

phosphoenolpyruvate	58114173	C00074	1.667	0.189	p<0.05	2.766	0.315	p<0.05
proline	145742	C00148	0.932	0.024	NS	0.619	0.072	p<0.05
pyrophosphate	1023	C00013	0.642	0.058	p<0.05	0.700	0.107	p<0.05
ribose-5-phosphate	439167	C00117	1.291	0.091	p<0.05	1.103	0.240	NS
sorbitol	5780	C00794	0.656	0.124	p<0.05	0.777	0.036	p<0.05
squalene	638072	C00751	0.615	0.107	p<0.05	0.844	0.296	NS
sucrose	5988	C00089	0.576	0.195	p<0.05	0.518	0.141	p<0.05
tagatose	439312	C00795	0.659	0.122	p<0.05	1.007	0.073	NS
UDP-glucuronic acid	17473	C00167	0.731	0.052	p<0.05	0.846	0.168	p<0.05
UDP-N-acetylglucosamine	445675	C00043	0.832	0.060	NS	0.684	0.071	p<0.05
uracil	1174	C00106	1.350	0.065	p<0.05	1.759	0.063	p<0.05
xylitol	6912	C00379	0.849	0.068	p<0.05	9.862	1.574	p<0.05

Supplementary Table 5. Non-significant metabolic changes assocated with increased oxidized glutathione. Related to Figure 6.

BinBase name	PubChem	KEGG	Fold change BSO V vehicle	SEM	P	Fold change Dia V vehicle	SEM	P
1-methylinosine NIST	65095		1.765	0.253	NS	0.813	0.156	NS
1-monoolein	5283468		1.598	0.194	NS	1.945	0.334	NS
1-monopalmitin	14900	C01885	1.063	0.189	NS	0.958	0.511	NS
1,3,5-trimethylcyanuric acid			0.849	0.058	NS	1.256	0.231	NS
2-hydroxyglutaric acid	43	C02630	0.906	0.019	NS	0.820	0.058	NS
2-hydroxyvaleric acid	98009		1.513	0.212	NS	1.630	0.281	NS
2-ketobutyric acid	58	C00109	1.441	0.175	NS	1.159	0.125	NS
2-monoolein	5319879		0.826	0.063	NS	0.823	0.121	NS
2-monopalmitin	123409		1.173	0.340	NS	3.179	0.497	NS
3-aminoisobutyric acid	64956	C05145	1.227	0.166	NS	1.326	0.349	NS
3-hydroxy-3-methylglutaric acid	1662	C03761	0.804	0.014	NS	1.133	0.064	NS
3-hydroxybutyric acid	92135	C01089	1.179	0.131	NS	1.148	0.375	NS
3,6-anhydro-D-galactose	16069996	C06474	0.848	0.099	NS	1.147	0.087	NS
4-aminobutyric acid	119	C00334	1.154	0.148	NS	0.383	0.048	NS
5'-deoxy-5'-methylthioadenosine	439176	C00170	0.837	0.058	NS	0.841	0.065	NS
acetophenone NIST	7410	C07113	1.040	0.049	NS	1.366	0.154	NS
aconitic acid	643757	C00417	1.047	0.104	NS	1.337	0.226	NS
adenine	190	C00147	0.859	0.024	NS	0.579	0.080	NS
alanine	5950	C00041	1.065	0.038	NS	0.888	0.086	NS
alanine-alanine	5484352	C00993	1.159	0.110	NS	1.114	0.074	NS
alpha-aminoadipic acid	92136	C00956	1.817	0.172	NS	0.769	0.063	NS
aminomalonate	100714	C00872	0.997	0.162	NS	0.663	0.142	NS
arachidic acid	10467	C06425	0.821	0.134	NS	0.912	0.182	NS
arachidonic acid	444899	C00219	1.319	0.116	NS	0.917	0.233	NS
asparagine	6267	C00152	1.305	0.088	NS	1.103	0.278	NS
beta-alanine	239	C00099	0.943	0.020	NS	1.153	0.175	NS
beta-glycerolphosphate	2526	C02979	0.790	0.094	NS	0.825	0.154	NS
cellobiose	6255	C01971	1.214	0.200	NS	1.707	0.339	NS
cholesterol	5997	C00187	0.959	0.113	NS	1.100	0.190	NS
cis-gondoic acid	5282768	C16526	0.992	0.077	NS	1.196	0.188	NS
citric acid	311	C00158	0.849	0.034	NS	1.136	0.077	NS
citrulline	9750	C00327	0.792	0.110	NS	1.028	0.032	NS
cystine	595	C01420	3.592	2.451	NS	0.979	0.159	NS
cytidine-5-monophosphate	6131	C00055	0.538	0.111	NS	1.155	0.514	NS
epsilon-caprolactam	7768	C06593	1.066	0.210	NS	1.388	0.264	NS

fructose-1-phosphate	439394	C01094	0.864	0.086	NS	1.017	0.346	NS
fructose-6-phosphate	440641	C05345	1.192	0.228	NS	1.983	0.694	NS
fumaric acid	444972	C00122	0.866	0.091	NS	0.762	0.110	NS
galactonic acid	128869	C00880	0.919	0.096	NS	0.963	0.212	NS
glucose-6-phosphate	5958	C00092	1.207	0.215	NS	2.970	0.767	NS
glutaric acid	743	C00489	0.998	0.134	NS	1.343	0.166	NS
glycerol	753	C00116	1.172	0.057	NS	1.012	0.107	NS
glycerol-alpha-phosphate	754	C03189	0.934	0.127	NS	1.064	0.382	NS
glycocyamine	763	C00581	1.036	0.197	NS	0.902	0.411	NS
glycolic acid	757	C00160	1.132	0.056	NS	1.413	0.418	NS
glycyl tyrosine	92829		0.936	0.143	NS	1.071	0.377	NS
heptadecanoic acid	10465		0.890	0.066	NS	1.144	0.177	NS
hexose-6-phosphate	208	C02965	1.097	0.253	NS	1.997	0.562	NS
histidine	6274	C00135	0.806	0.038	NS	0.801	0.097	NS
hydroquinone	785	C00530	0.965	0.076	NS	1.378	0.155	NS
hydroxycarbamate NIST	16639161		1.169	0.185	NS	1.849	0.547	NS
hydroxylamine	787	C00192	1.265	0.210	NS	1.889	0.493	NS
hypoxanthine	790	C00262	1.128	0.061	NS	1.111	0.030	NS
ile-ile NIST			0.616	0.093	NS	1.596	0.515	NS
inosine 5'-monophosphate	8582	C00130	0.658	0.064	NS	0.863	0.058	NS
inositol-4-monophosphate	440043	C03546	1.020	0.073	NS	1.206	0.358	NS
isohexonic acid	604		1.129	0.048	NS	1.336	0.116	NS
isoleucine	6306	C00407	1.060	0.054	NS	1.157	0.126	NS
isomaltose	439193	C00252	0.912	0.145	NS	1.156	0.211	NS
L-DOPA			1.100	0.107	NS	0.944	0.436	NS
lactamide	94220		1.112	0.128	NS	1.139	0.129	NS
lactitol	157355	C13542	1.371	0.140	NS	1.405	0.512	NS
lanosterol	246983	C01724	0.876	0.081	NS	1.110	0.312	NS
leucine	6106	C00123	0.998	0.090	NS	1.195	0.092	NS
levoglucosan	2724705		1.575	1.083	NS	1.693	1.414	NS
lysine	5962	C00047	1.111	0.160	NS	1.000	0.239	NS
maleimide	10935	C07272	1.095	0.074	NS	1.549	0.132	NS
maltose	439186	C00208	0.920	0.140	NS	1.467	0.232	NS
mannose	18950	C00159	0.803	0.081	NS	0.799	0.104	NS
methanolphosphate	13130		1.027	0.085	NS	0.887	0.094	NS
methionine	6137	C00073	0.951	0.022	NS	0.973	0.052	NS
methionine sulfoxide	158980	C02989	0.915	0.030	NS	0.870	0.112	NS
methylhexose nist	560150		1.416	0.111	NS	1.850	0.435	NS
myristic acid	11005	C06424	0.915	0.115	NS	1.274	0.217	NS
N-acetylglutamate	70914	C00624	0.823	0.034	NS	0.838	0.100	NS
N-acetylmannosamine	439281	C00645	1.028	0.083	NS	0.932	0.062	NS

nicotinamide	936	C00153	0.915	0.050	NS	1.007	0.056	NS
oleic acid	445639	C00712	1.355	0.208	NS	1.068	0.426	NS
ornithine	6262	C00077	1.091	0.074	NS	0.934	0.141	NS
palmitic acid	985	C00249	0.864	0.059	NS	1.090	0.069	NS
pelargonic acid	8158	C01601	0.988	0.078	NS	1.117	0.108	NS
phenylalanine	6140	C00079	0.897	0.027	NS	0.924	0.026	NS
phosphate	1004	C00009	1.025	0.035	NS	1.006	0.058	NS
phosphoethanolamine	1015	C00346	1.212	0.249	NS	0.806	0.194	NS
phosphogluconic acid	91493	C00345	1.103	0.087	NS	1.114	0.326	NS
putrescine	1045	C00138	1.158	0.102	NS	0.849	0.144	NS
pyruvic acid	1060	C00022	1.000	0.059	NS	1.396	0.109	NS
ribose	5779	C00121	0.960	0.080	NS	1.119	0.319	NS
serine	5951	C00065	1.144	0.077	NS	0.982	0.090	NS
spermidine	1102	C00315	1.253	0.139	NS	0.821	0.256	NS
stearic acid	5281	C01530	0.883	0.079	NS	1.033	0.121	NS
taurine	1123	C00245	0.851	0.175	NS	0.828	0.296	NS
threonine	6288	C00188	0.954	0.102	NS	1.060	0.099	NS
tocopherol alpha-	638015	C00376	0.804	0.066	NS	0.851	0.099	NS
tryptophan	6305	C00078	0.833	0.032	NS	0.876	0.019	NS
tyrosine	6057	C00082	0.812	0.032	NS	0.877	0.041	NS
urea	1176	C00086	0.827	0.082	NS	1.063	0.146	NS
uridine	6029	C00299	1.533	0.243	NS	0.966	0.185	NS
valine	6287	C00183	1.086	0.049	NS	1.190	0.115	NS
xanthine	1188	C00385	0.986	0.060	NS	0.896	0.235	NS
xanthosine	64959	C01762	0.947	0.093	NS	1.315	0.260	NS
zymosterol	92746	C05437	0.910	0.090	NS	0.822	0.130	NS

Supplementary Figure 1. Related to Figure 1.

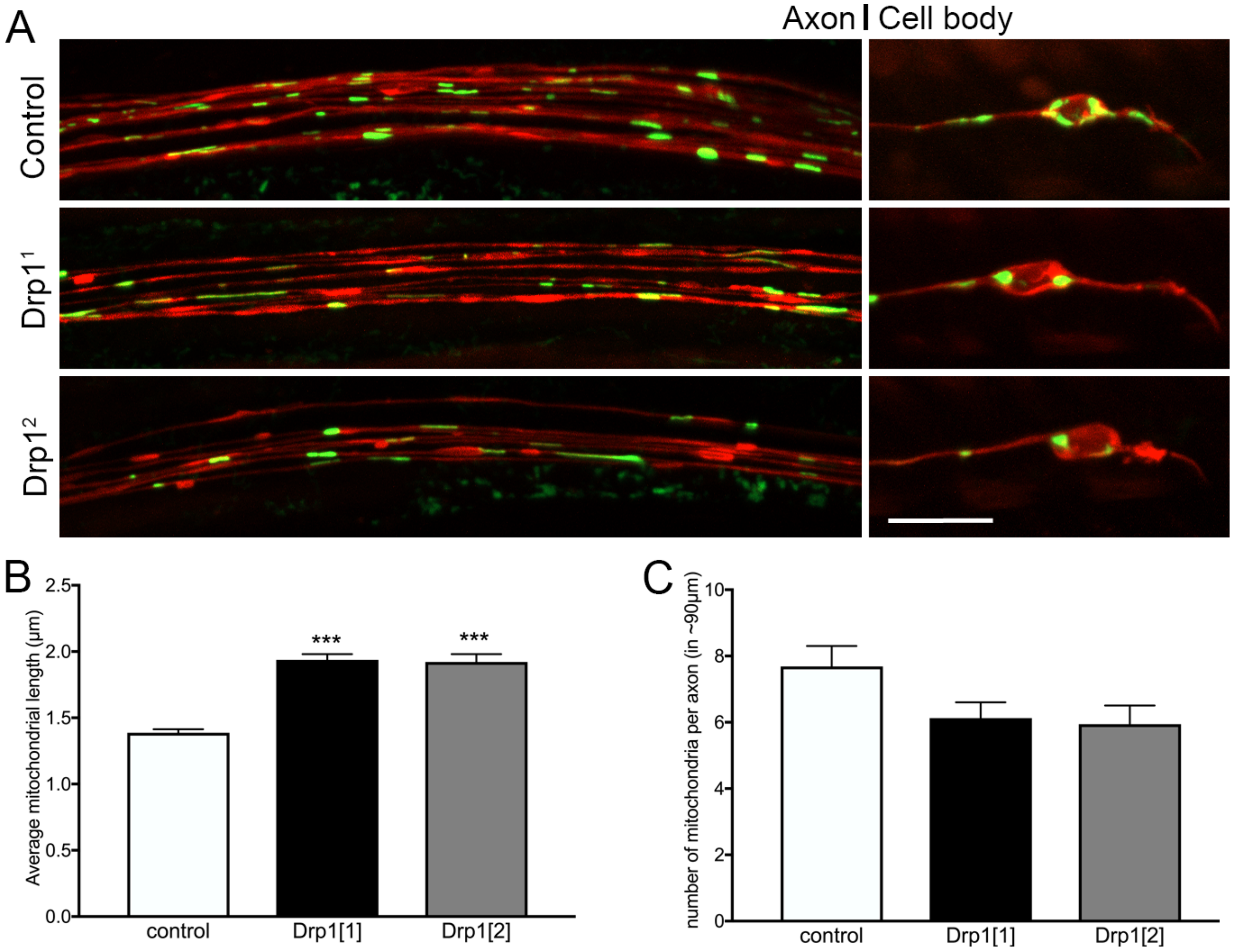

Supplementary Figure 1. Drp1 causes enhanced mitochondrial fusion in adult neurons *in vivo.* A) Two independent Drp1 alleles were used as positive controls for the unbiased genetic screen. Compared to controls mitochondria appeared fused in the axon and hyperfused in the cell body. B) Quantification shows that mitochondria were significantly longer in axon stretches. C) Increased mitochondrial length was not associated with a change in mitochondrial number. Data was analyzed by 1-way ANOVA and significant differences compared to control annotated as p<0.001***. Graphs are expressed as Mean ± SEM and N= ≥10 wings for each group. Scale bar = 10µm.

Supplementary Figure 2. Related to Figure 2.

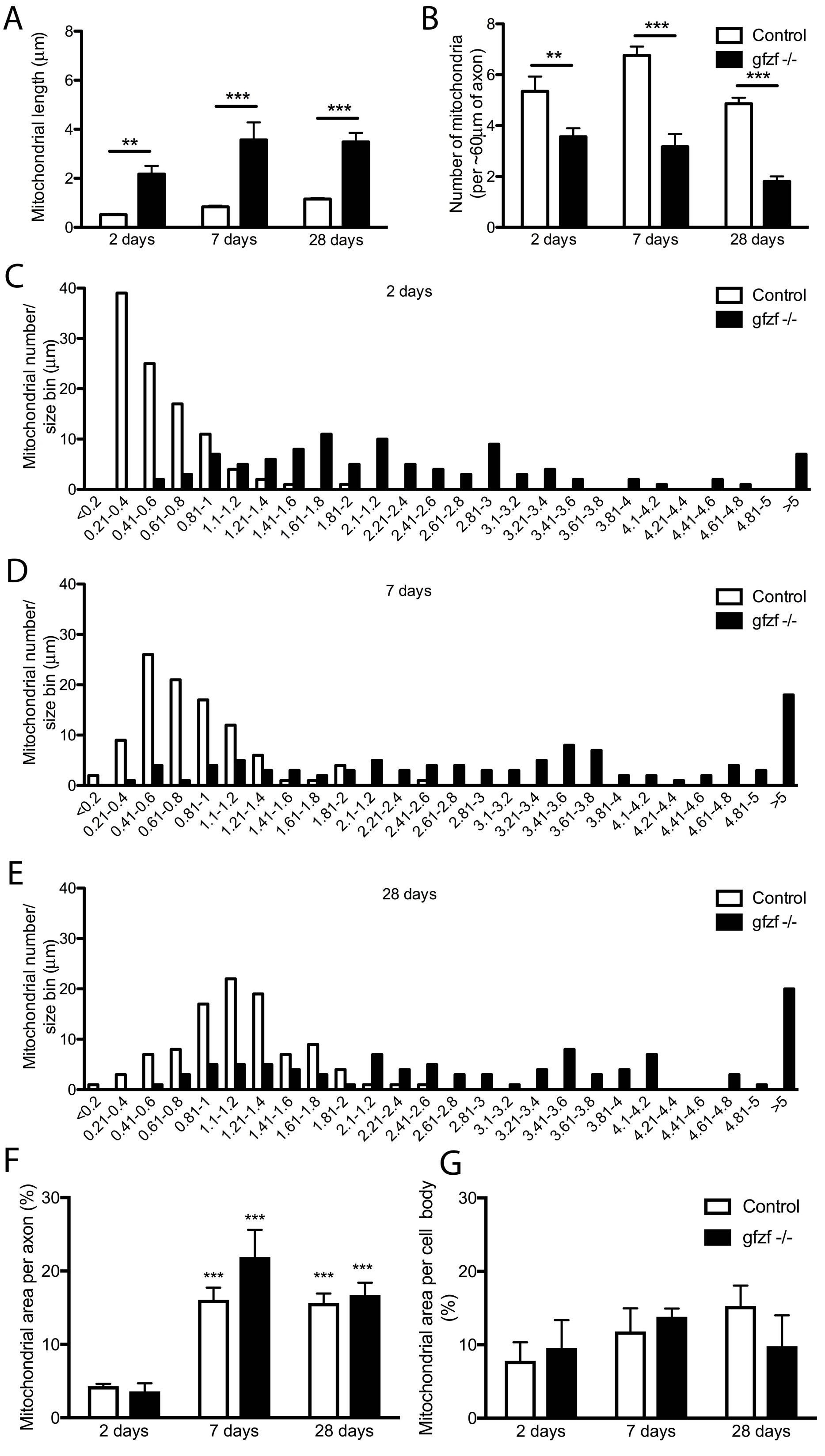

Supplementary Figure 2. Mutations in a novel Glutathione-S-transferase gene, *gfzf*, causes an increase in mitochondrial length throughout the length of the axon. A) The increase in mitochondrial length compared to control was further observed at all ages when analyzed in distal axons stretches that are proximal to the cells bodies. B) An age dependent reduction in mitochondrial number was also observed in distal region of the axon, where mitochondrial number was found to be marginally reduced at 2 days p.e. and greatly reduced at both 7 and 14 days p.e. C) Analyzing the distribution of mitochondria lengths, by size bin, in the distal axons at 2 days, revealed that there was a significant shift in *gfzf*-/- clones to lengths of ≥2.1, with no control mitochondrial reaching these lengths. D) The frequency distribution of mitochondrial length by size bins is further shifted in *gfzf* mutant clones at 7 days p.e. towards lengths of ≥2.61 E) At 28 days p.e. the distribution of mitochondrial in *gfzf* mutant clones are similarly shifted to greater size bins groups in distal axons, compared to control. F) The % area occupied with mitochondria within a 50μm axonal stretch was found to increase from 2-7 days regardless of genotype. G) % area occupied with mitochondria within the cell body was not significantly altered. Data was analyzed by 2-way ANOVA and significant differences annotated as $p<0.01$**, $p<0.001$***. Graphs are expressed as Mean ± SEM and N= ≥10 wings for each group.

Supplementary Figure 3. Related to Figure 3.

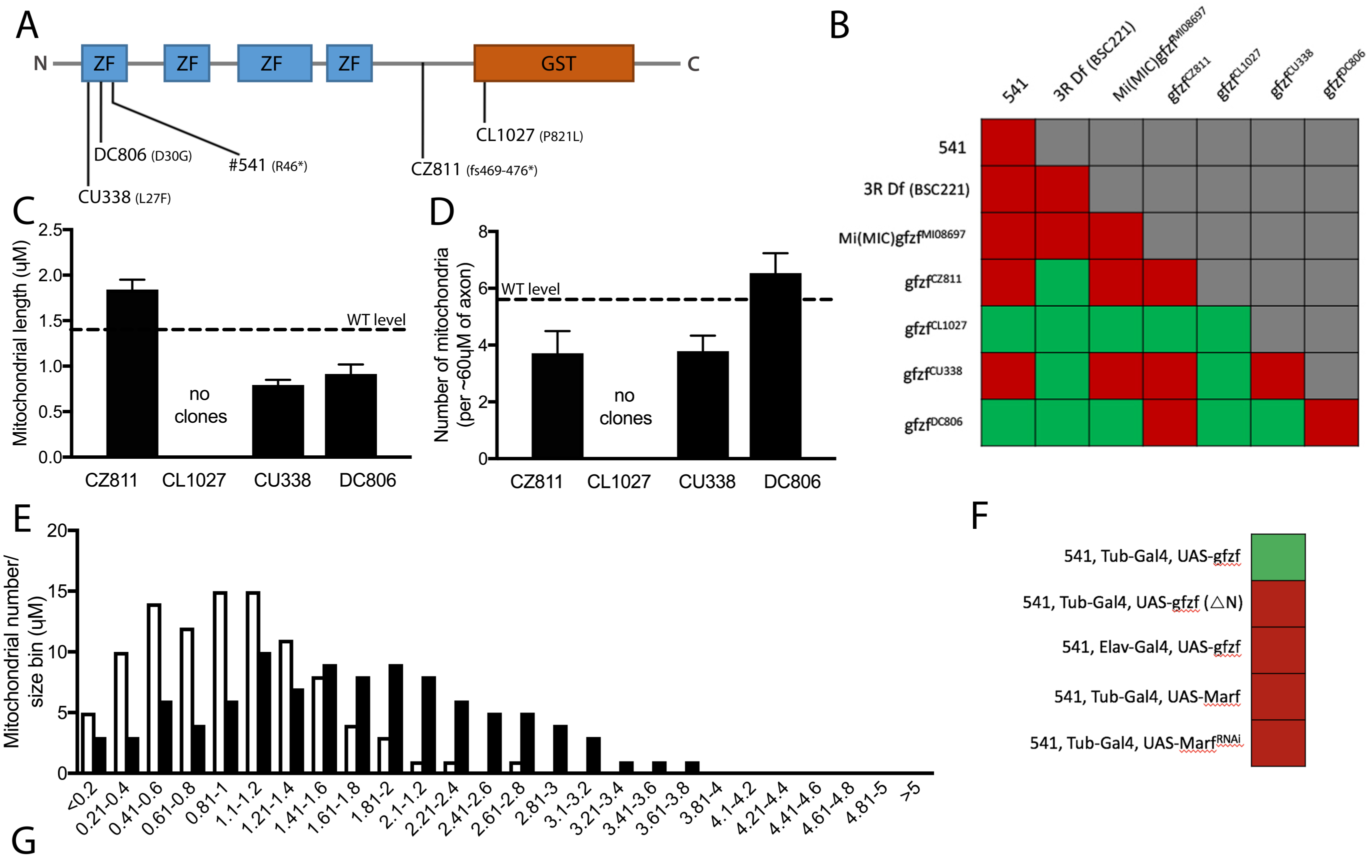

G

```
gfzf    MKLYAVSDGPPSLAVRMTLKALDIQYQLINVDFCAMEHRSEEYSKMNPQF-NMGFYYAP ISA - - HS- MAPI FFDYKRTPMSLKKVQNALDVFETYLQRKE IPVLDDDGFYLSESIAIMQYLCDKYAPDSTLYPQDVNVRAVINQRLCLGTKYA- - - - - -AGENITIADFALIS
        ++LY          P  AV +    K   DI ++L   VD       +H S+  ++++NP  + +         + + A    H  M   P+F        +P  +L          LDV      LQ  K++P  L  D     F L+ES+AI+    YL    KY          YPQD+     RA   +++ L  L    K+               G  +I++AD    I+
GSTT1   L ELYLDL LSQPCRAVY IFAKKNDIPFE LR IVDL I KGQHLSDAFAQVNPLWQHTTLRRSCLRALWHKVMFPVFLGEPVSPQT LAATLAE LDVT- - -LQL KKVPALKDGDFTLTESVAILLYLTRKYKVPDYWYPQDLQARARVDEYLA LEDKFLQNKAF LTGPHISLADLVAIT
```

Supplementary Figure 3. Analysis of other *gfzf* alleles and alignment with human GSTT1. A) A schematic diagram of *gfzf* is outlined showing the four zinc finger domains and GST domain. A map of known mutations within this gene are indicated by their relative position compared to the mutation discovered in the mitochondria screen (#541). B) Cross complementation analysis indicates that #541 is likely to be the most sever mutation, as other mutations are viable (green) when crossed to either a deficiency line (BSC221), a Mi(MIC) line (*gfzf*^{MI08697}) or both, whereas #541 remains lethal (red) when crossed with the same lines. C) Homozygous MARCM neuronal clones were generated for each available allele and mutations annotated as *gfzf*^{CZ811}, *gfzf*^{CU338} or *gfzf*^{DC806} failed to show significantly increased mitochondrial lengths at 7 days p.e. compared to control, as indicated by the dashed line. The allele harboring the mutation annotated *gfzf*^{CL1027} failed to show any clones and could not be quantified. D) Mutations annotated as *gfzf*^{CZ811} and *gfzf*^{CU338} caused a reduction in mitochondrial numbers in long axon stretches compared to control, as indicated by the dashed line. E) When separated by size bin, mitochondrial residing in *gfzf*^{CZ811} mutant clones was shifted toward increased length compared to control. Fewer short mitochondria were seen at ranges from <0.2μm to 1.2-1.4μm and increased longer mitochondria observed at ranges from 1.61-1.8μm to 3.61-3.8μm. F) Rescue experiments indicate that lethality is the result of *gfzf* loss in other non-neuronal tissues. G) A alignment of the GST domain of *gfzf* with human GSTT1. Graphs are expressed as Mean ± SEM and N= ≥10 wings for each group.

Supplementary Figure 4. Related to Figure 3.

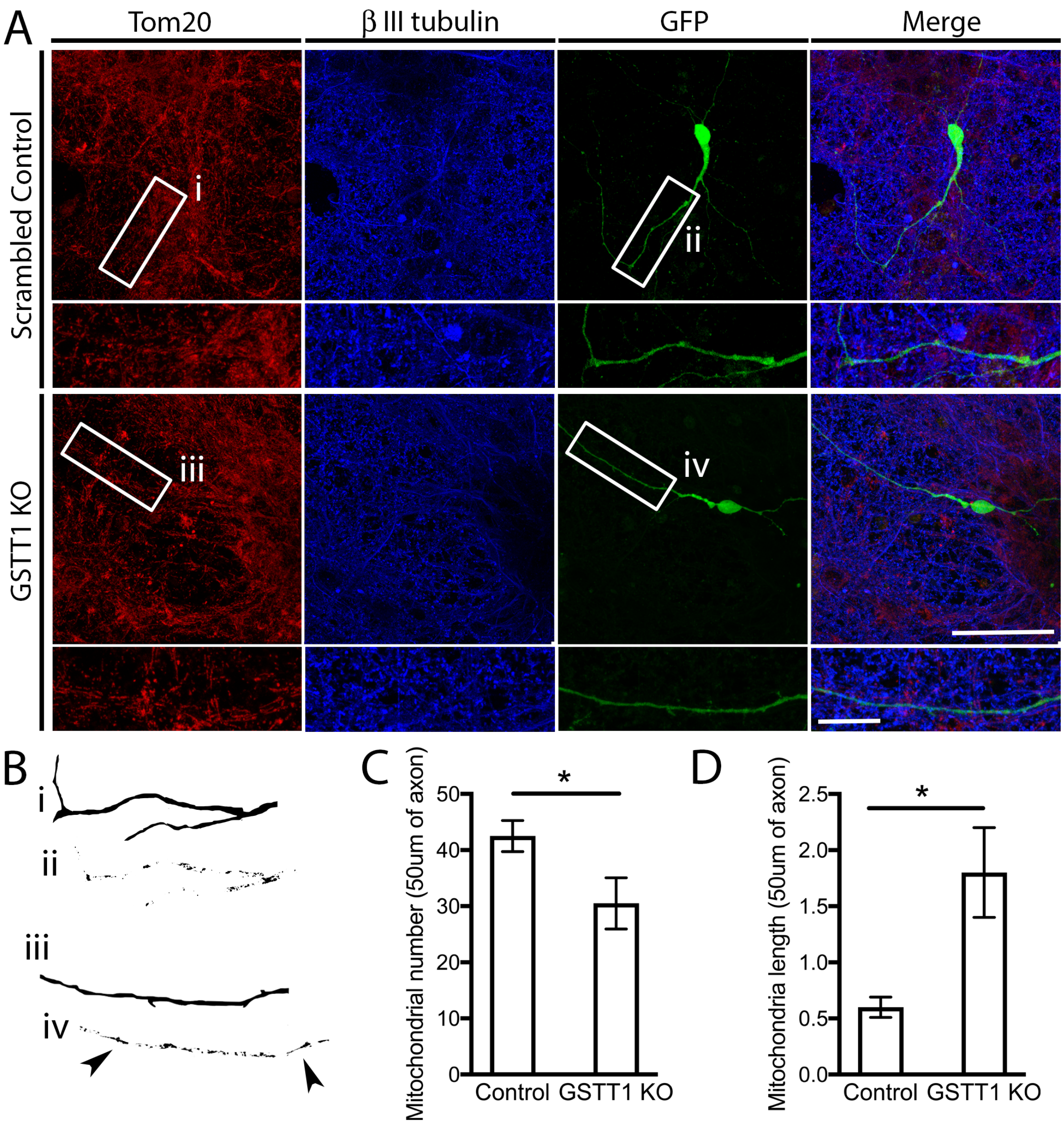

Supplementary Figure 4. gRNA/ Cas9 mediated knockout of GSTT1 in neurons *in vitro* causes increased mitochondrial length. A) Neuronal primary cultures were transfected with gRNA/Cas9 Knockout (KO) plasmids using sequences targeting mouse GSTT1 or a scrambled control and allowed to mature until 3 weeks. Following fixation neurons and mitochondria were visualized using antibodies against βII tubulin (blue) and Tom20 (red). Transfected cells were labelled with GFP. B) Using the GFP as reference a mask was created across a 40μm neuronal projection and the mitochondria contained analyzed using ImageJ. GSTT1 ablation caused an increased number of larger mitochondria as indicated by the arrows. C) Quantification shows that the number of mitochondria was reduced in GSTT1 mutants. D) Average mitochondrial length was increased. Data was analyzed by unpaired T-test and significant differences annotated as $p<0.05$* between genotypes. Graphs are expressed as Mean ± SEM and N= 4 for each group. Scale bars =40μm (top panels), =10 μm (bottom panels).

Supplementary Figure 5. Related to Figure 3.

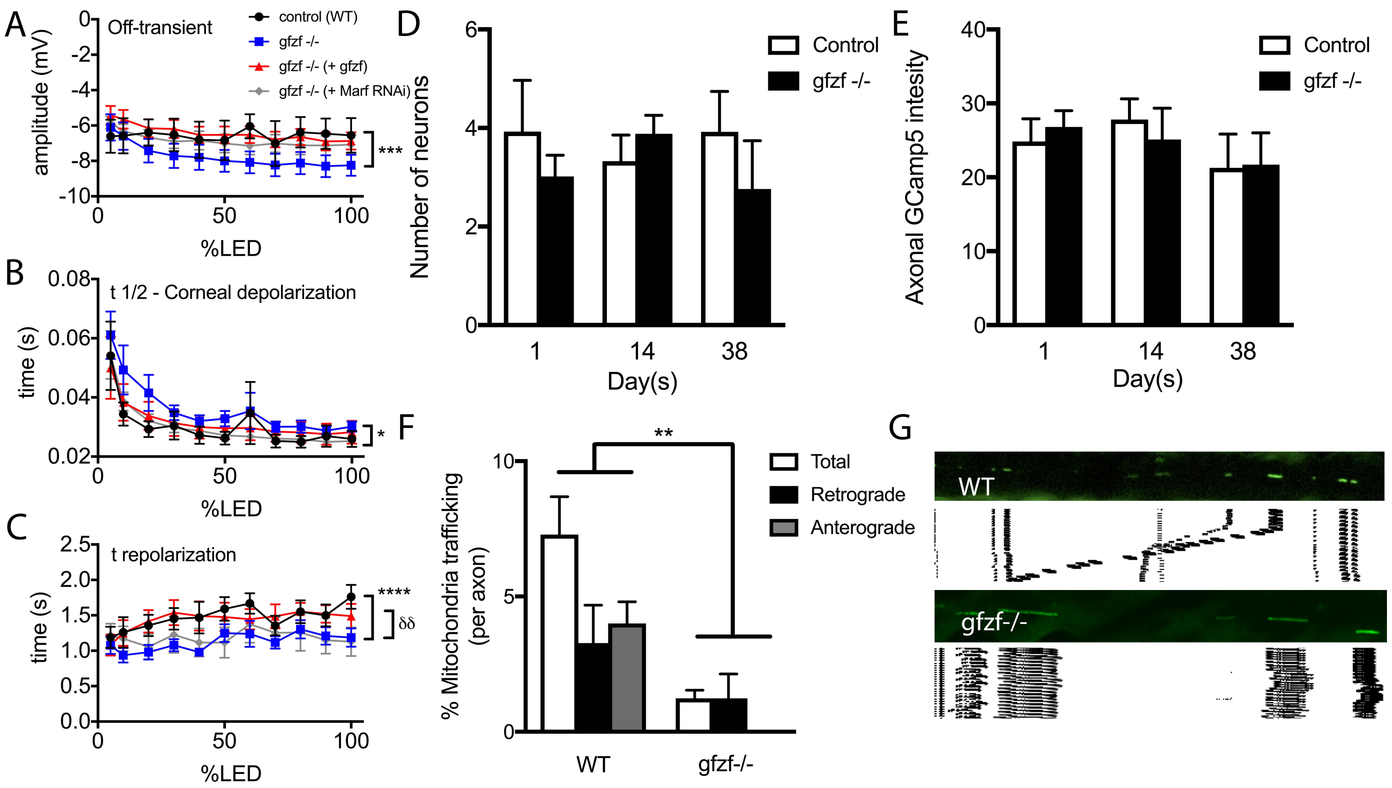

Supplementary Figure 5. Gfzf loss results in physiological changes and mitochondrial trafficking deficits. Electroretinogram (ERG) recordings were conducted at a range of LED intensities in aged flies at 28 days p.e. A) Significant changes in the Off-transient seen between WT and *gfzf-/-* mutant backgrounds could be rescued by re-expression of Gfzf and by RNAi mediated knockdown of Marf. B) The half-time to corneal depolarization was also rescued by both approaches. C) Time to repolarization was rescued fully by re-expression of Gfzf and partially by Marf knockdown. D) The number of neurons in *gfzf*$^{-/-}$ mutants is not significantly different compared to WT when quantified at young and old age. E) Baseline Ca^{2+} levels were also not changed overtime. F) Gfzf loss causes a significant decrease in axonal mitochondrial transport in both the retrograde and anterograde directions at 7 days p.e. G) Representative kymographs of mitochondrial transport. Significant differences between *gfzf-/-* and control were annotated as p<0.05*, p<0.001*** & p<0.0001**** and *gfzf-/-* and *gfzf-/-* (+ Marf RNAi) as p<0.01$^{\delta\delta}$. Graphs are expressed as Mean ± SEM and N=4-6 flies used for each group.

Supplementary Figure 6. Related to Figure 3.

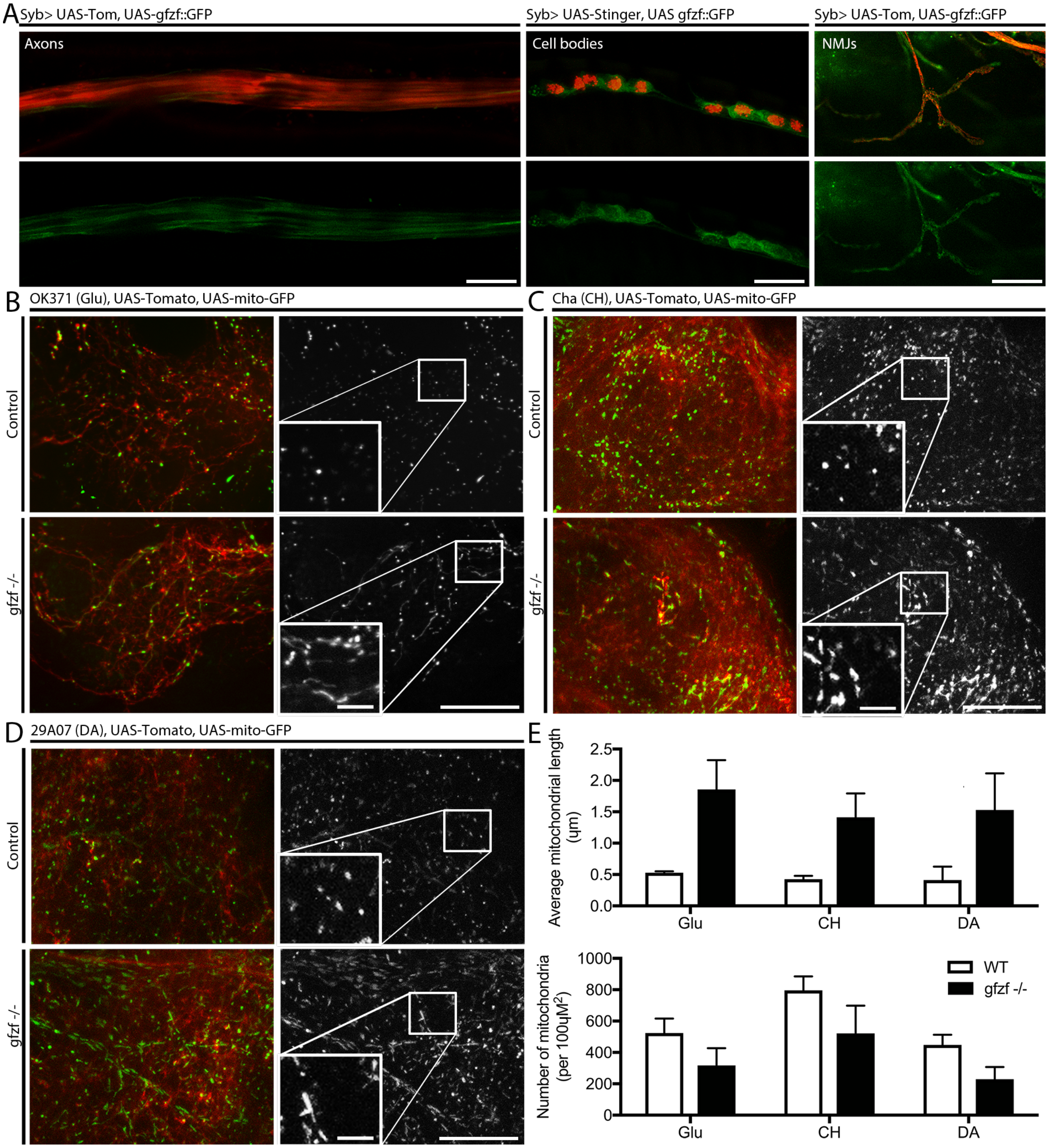

Supplementary Figure 6. Gfzf is ubiquitously expressed in neurons and regulates mitochondrial morphology in different neuronal subtypes within the adult CNS. A) Gfzf tagged with GFP on the N-terminus (green) was found to ubiquitously expressed within neurons (red) when driven by the synapsin promotor, yet was weakly expressed in neuronal nuclei, as outlined by expression of red stinger. Specifically, Gfzf::GFP was expressed within axons (left), cell bodies (middle) and at the neuromuscular junction (right). B-D) Mitochondria length was increased in the terminals of glutamatergic (Glu), cholinergic (Cha) and dopaminergic (DA) neuron *gfzf*-/- MARCM clones, at 7 days p.e., compared to respective controls. E) Quantification of these terminal regions within the CNS revealed that mitochondria length is increased and mitochondrial number reduced in all neuronal subtypes analyzed. Graphs are expressed as Mean ± SEM and N= ≥5 brains for each group, where 3 images were averaged for each animal. Scale bars =10 μm (top left), =20μm (top middle & top right), =100μm (bottom) = 3μm (bottom insets).

Supplementary Figure 7. Related to Figure 3.

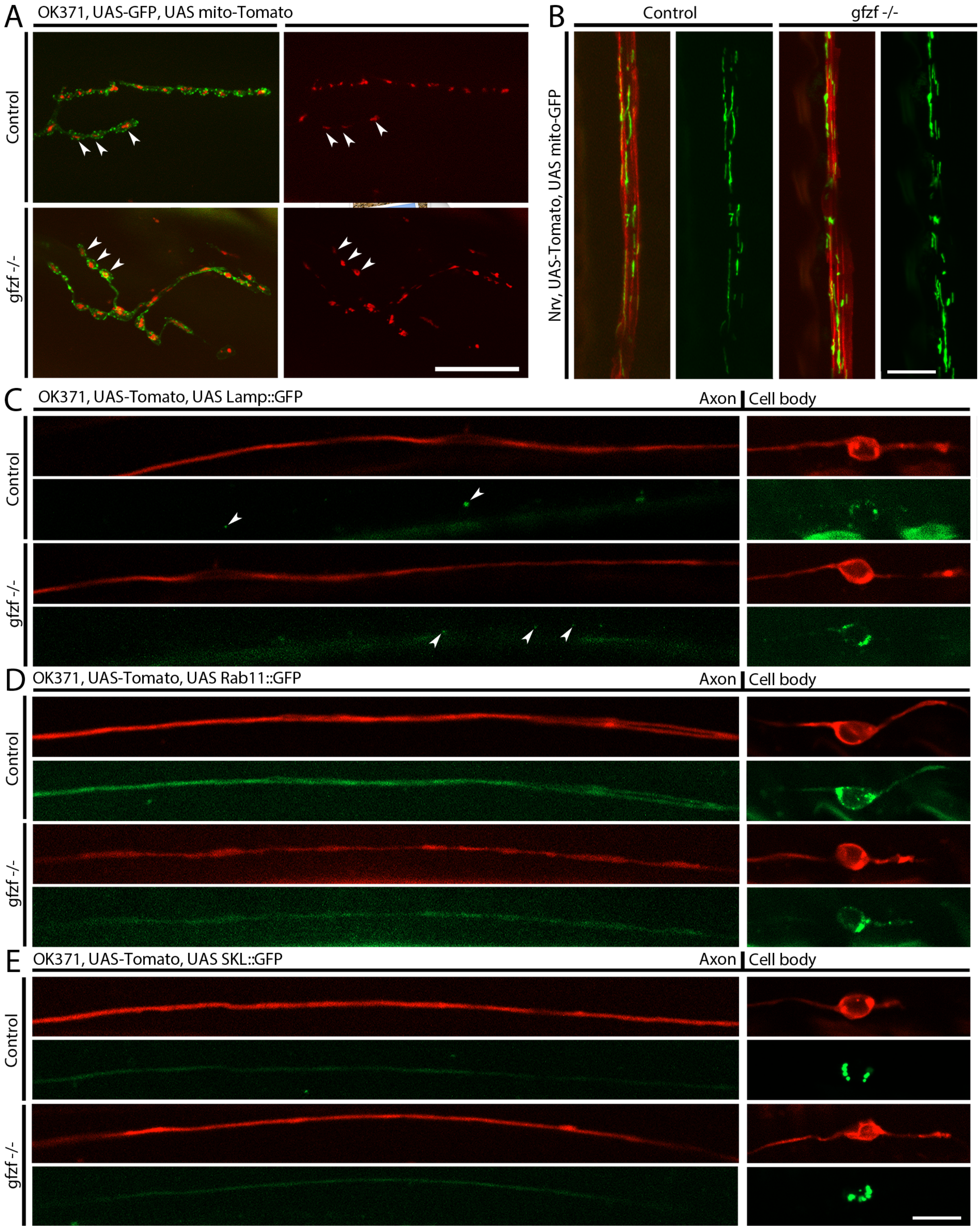

Supplementary Figure 7. *gfzf* ablation did not affect mitochondrial morphology at the neuromuscular junction or in wrapping glia cells and does not affect other vesicles. A) mitochondrial distribution at the neuromuscular junction was found to coincide with synaptic boutons and no observable difference in mitochondrial morphology could be detected between *gfzf* mutant clones and age matched controls at 7 days p.e. B) There was also no difference in mitochondrial morphology and distribution in wrapping glia clones induced in the adult wing. C-E) Similarly, the intensity, morphology and distribution of lysosomes, endosomes and peroxisomes was not found to be altered in *gfzf* mutant clones compared to control clones, as visualized by Lamp1::GFP, Rab11::GFP and SKL::GFP respectively at 7 days p.e. Scale bars =20μm (top left), =10 μm (top right & bottom). Arrows indicate individual vesicles in axon stretches.
